# Supplementary material for: Maize Phyllosphere Microbial Community Niche Development Across Stages of Host Leaf Growth
Source: F1000Res. 2018 Jan 18;6:1698. Originally published 2017 Sep 18. [Version 3] doi: 10.12688/f1000research.12490.3 (PMC5861518; doi:10.12688/f1000research.12490.3)

Supplemental Figure S1 a-n Arisa Plots of Significant Co-occurring OTU Pairs

Fig. S1a) day 30 stacked plot

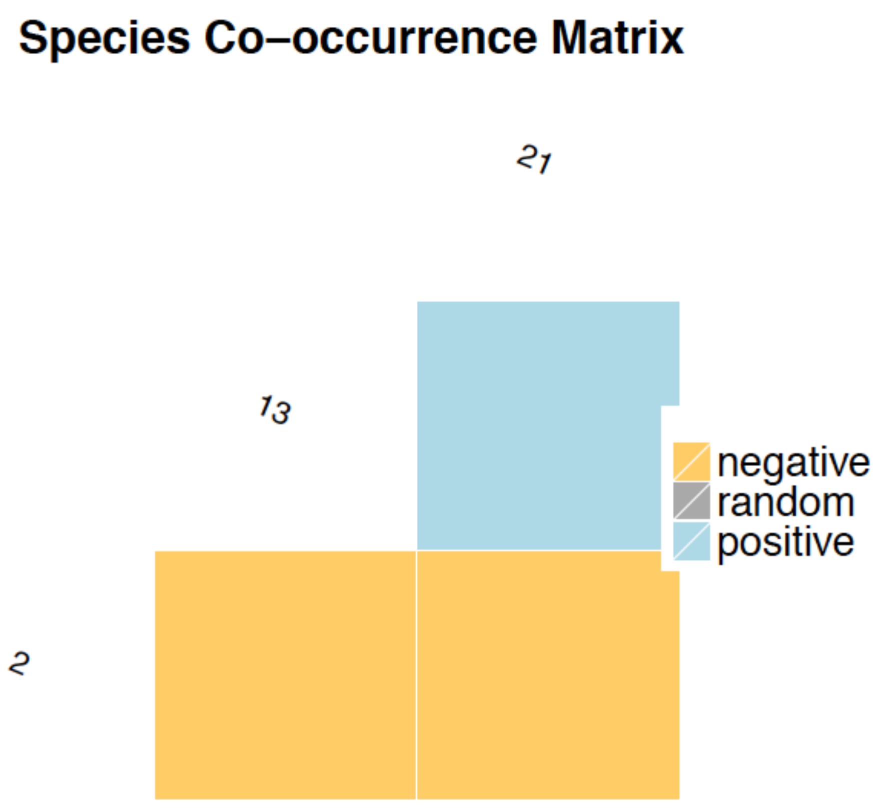

Fig. S1b) Stacked plot stage 41

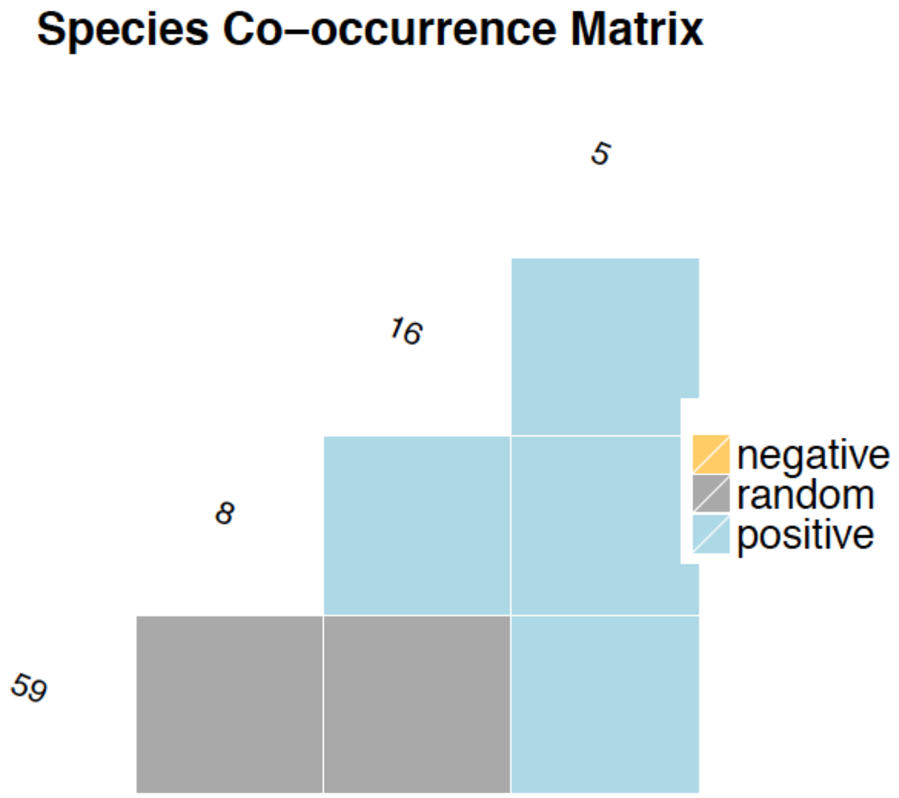

Fig. S1c) Stacked plot stage 48

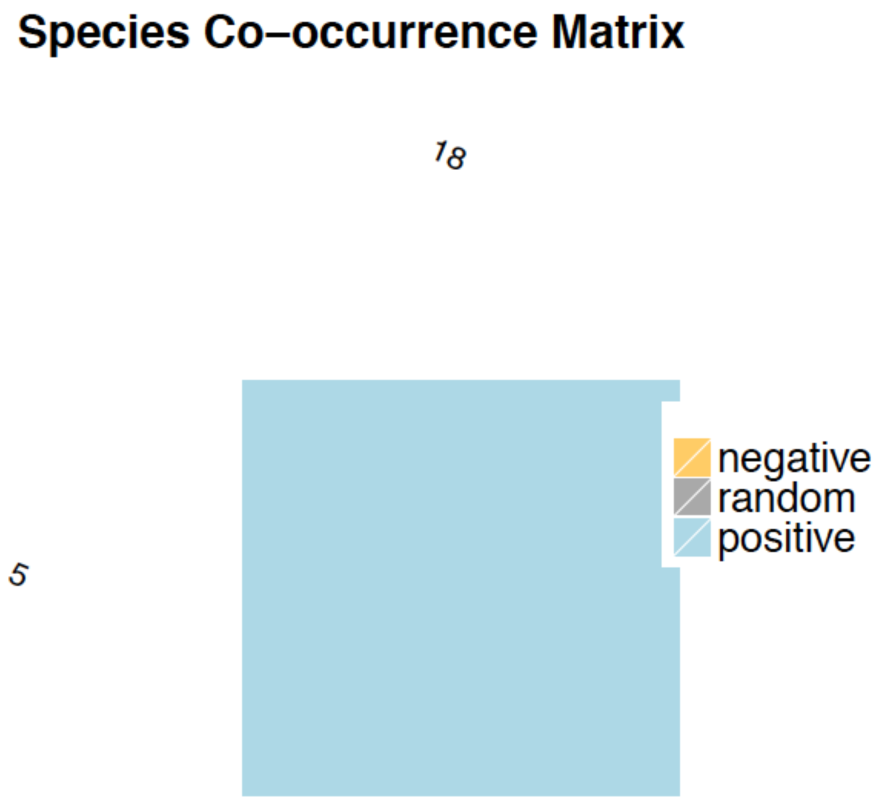

Fig. S1d) Stacked plot stage 58
(Note—no pairs were significant at this stage)

Species Co–occurrence Matrix

Fig. S1e) Stacked plot stage 62

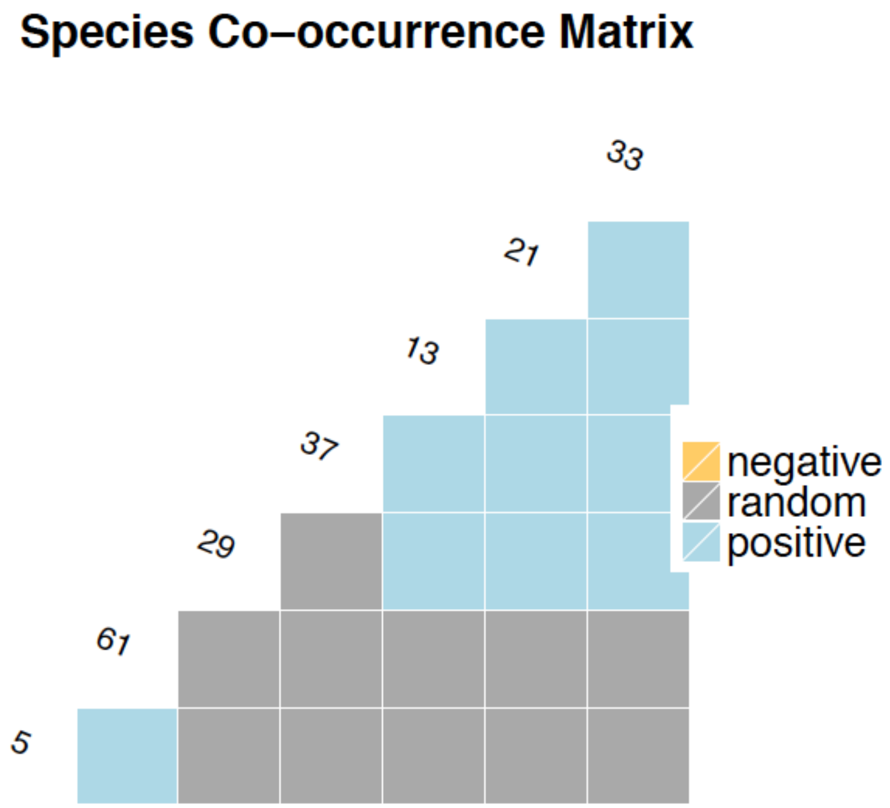

Fig. S1f) Stacked plot stage 72

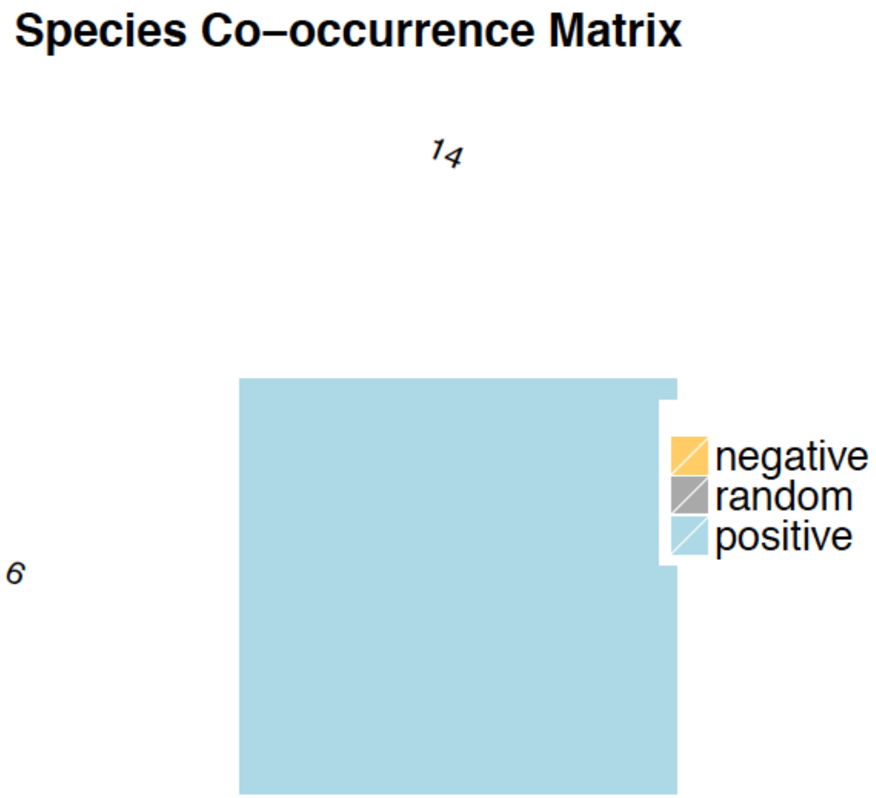

Fig. S1g) Stacked plot stage 80

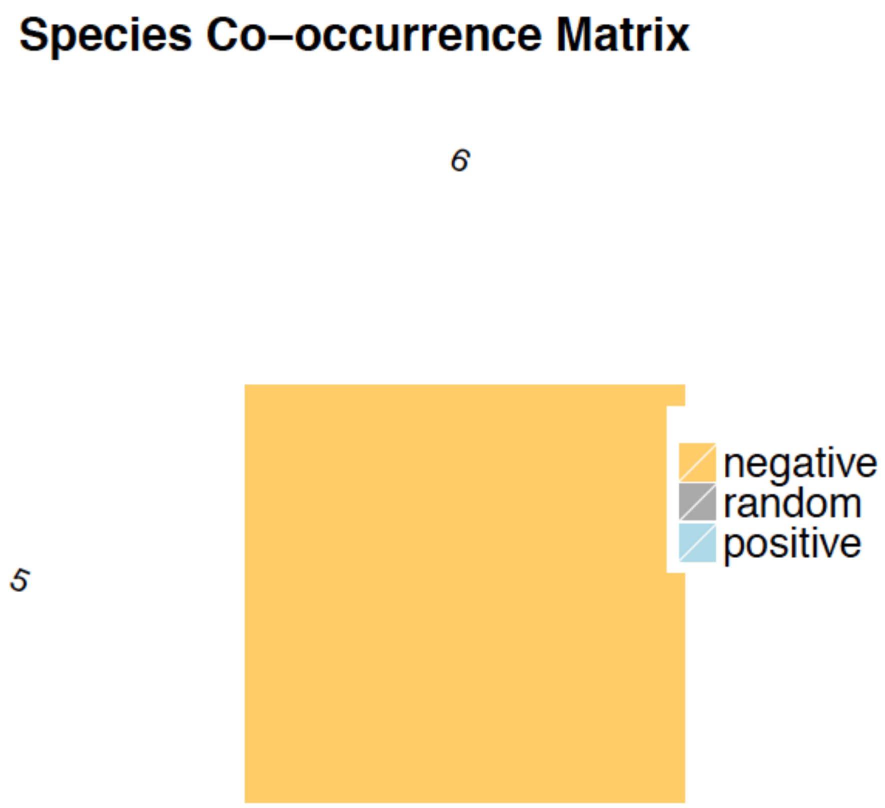

Fig. S1h) Paired OTU plot stage 30

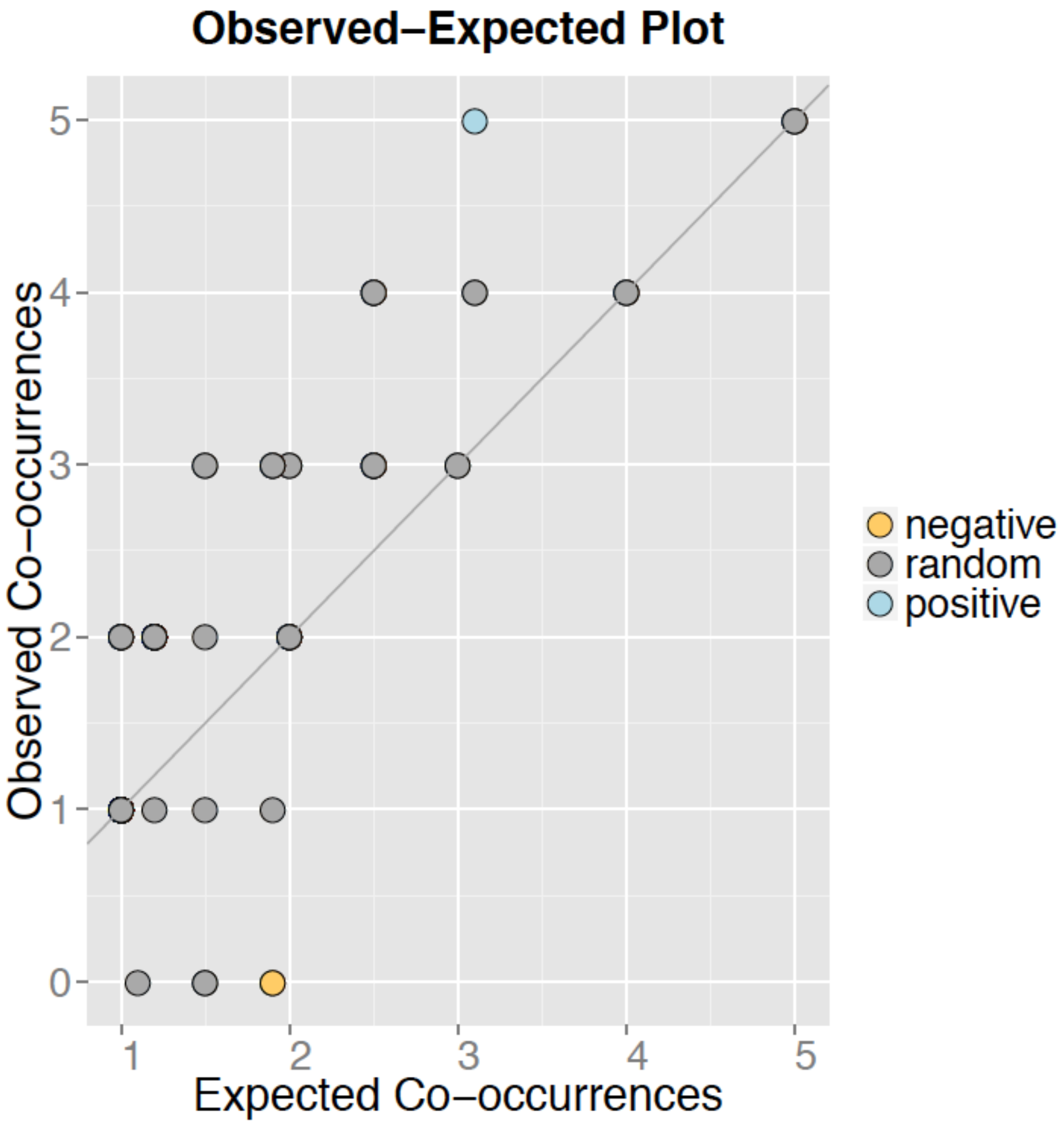

Fig. S1i) Paired OTU plot stage 41

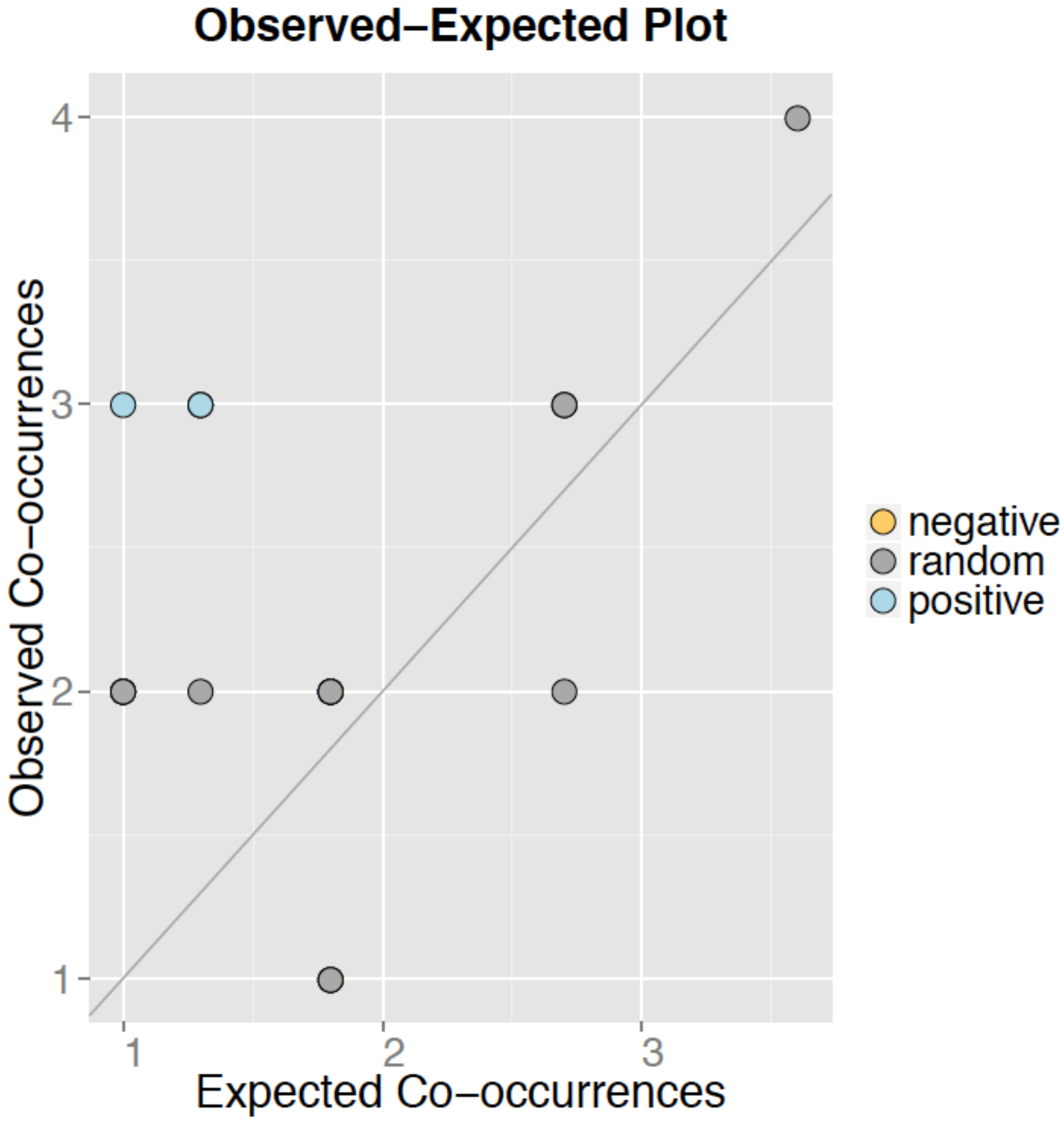

Fig. S1j) Paired OTU plot stage 48

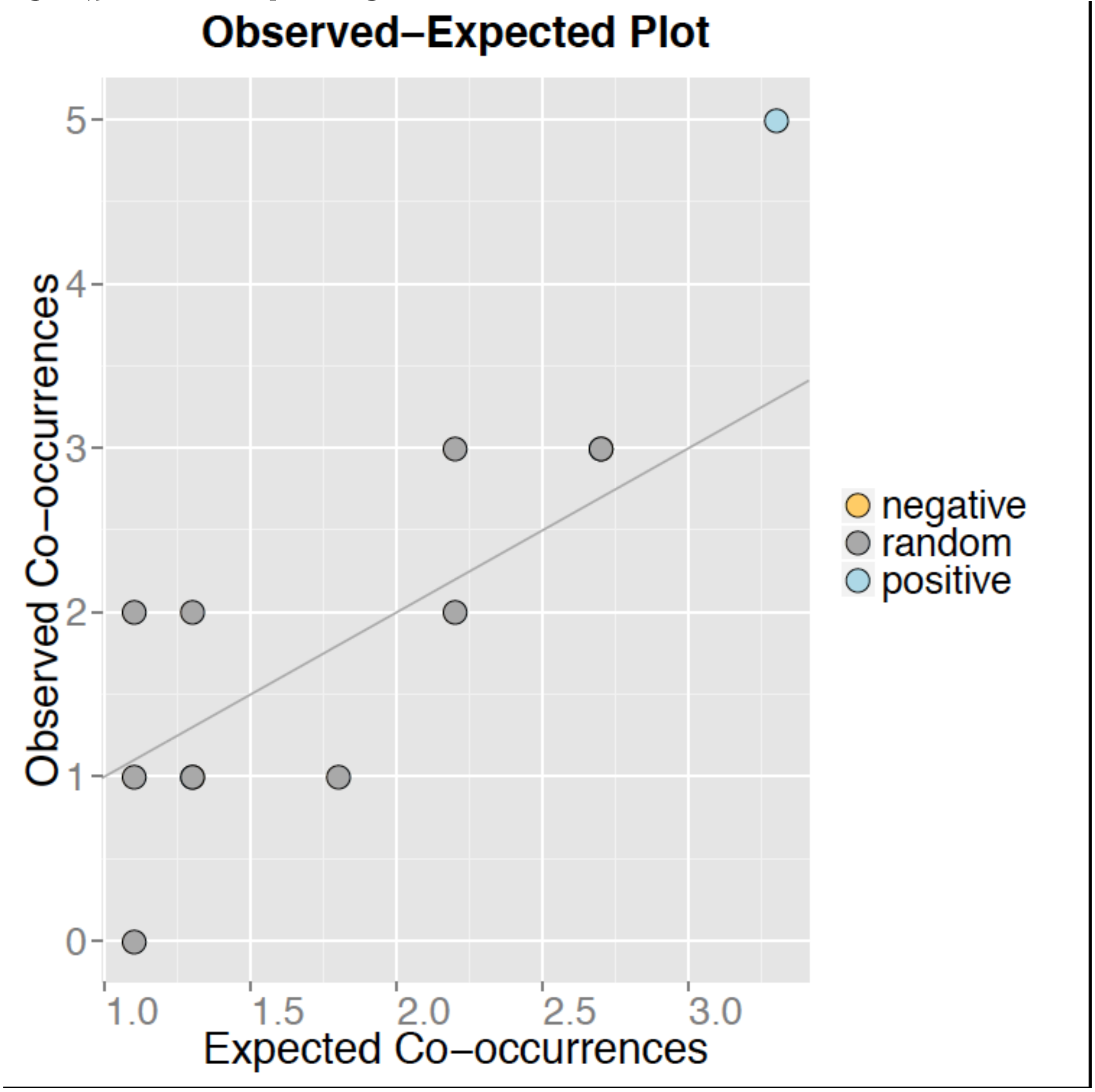

Fig. S1k) Paired OTU plot stage 58

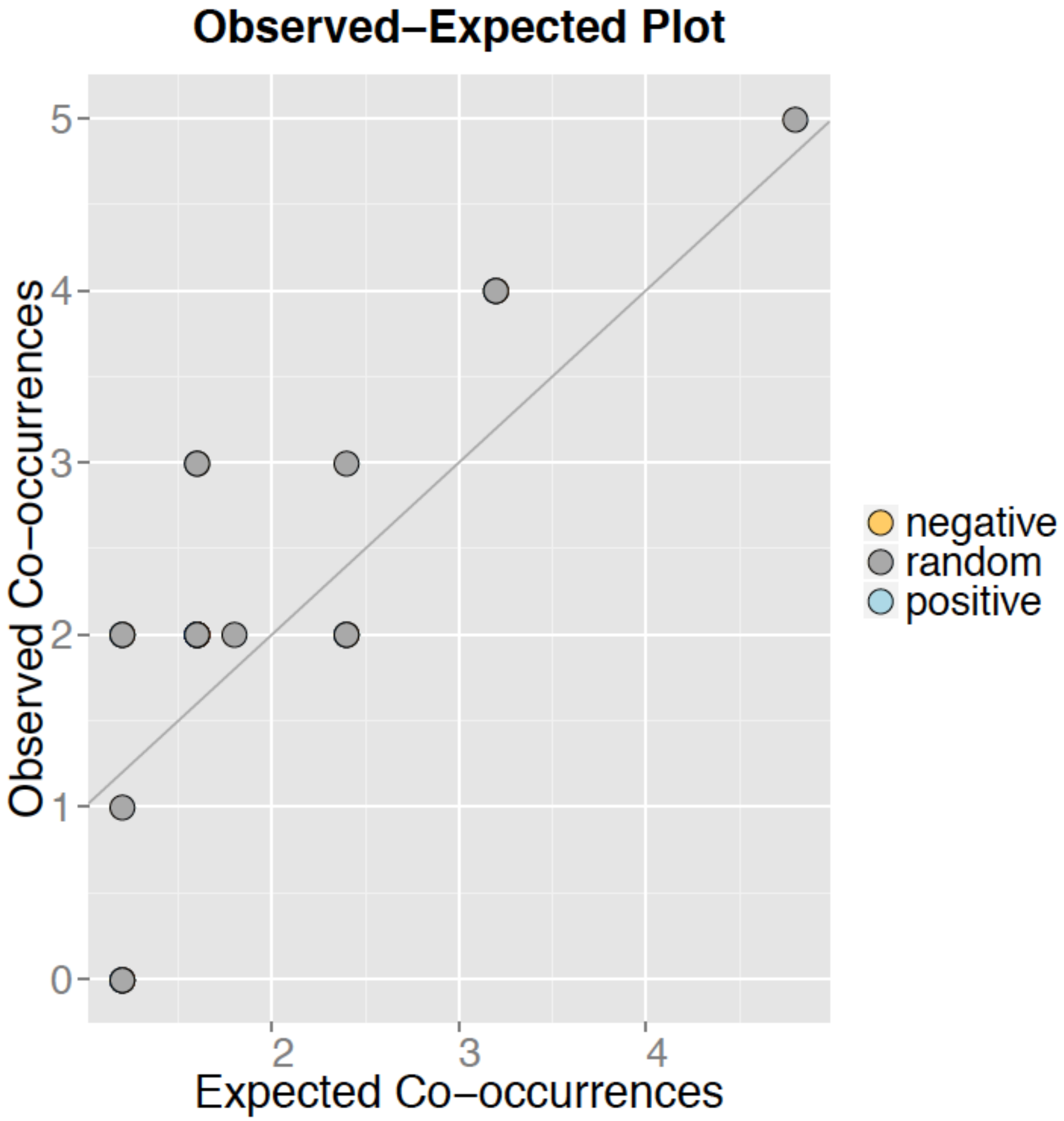

Fig. S1l) Paired OTU plot stage 62

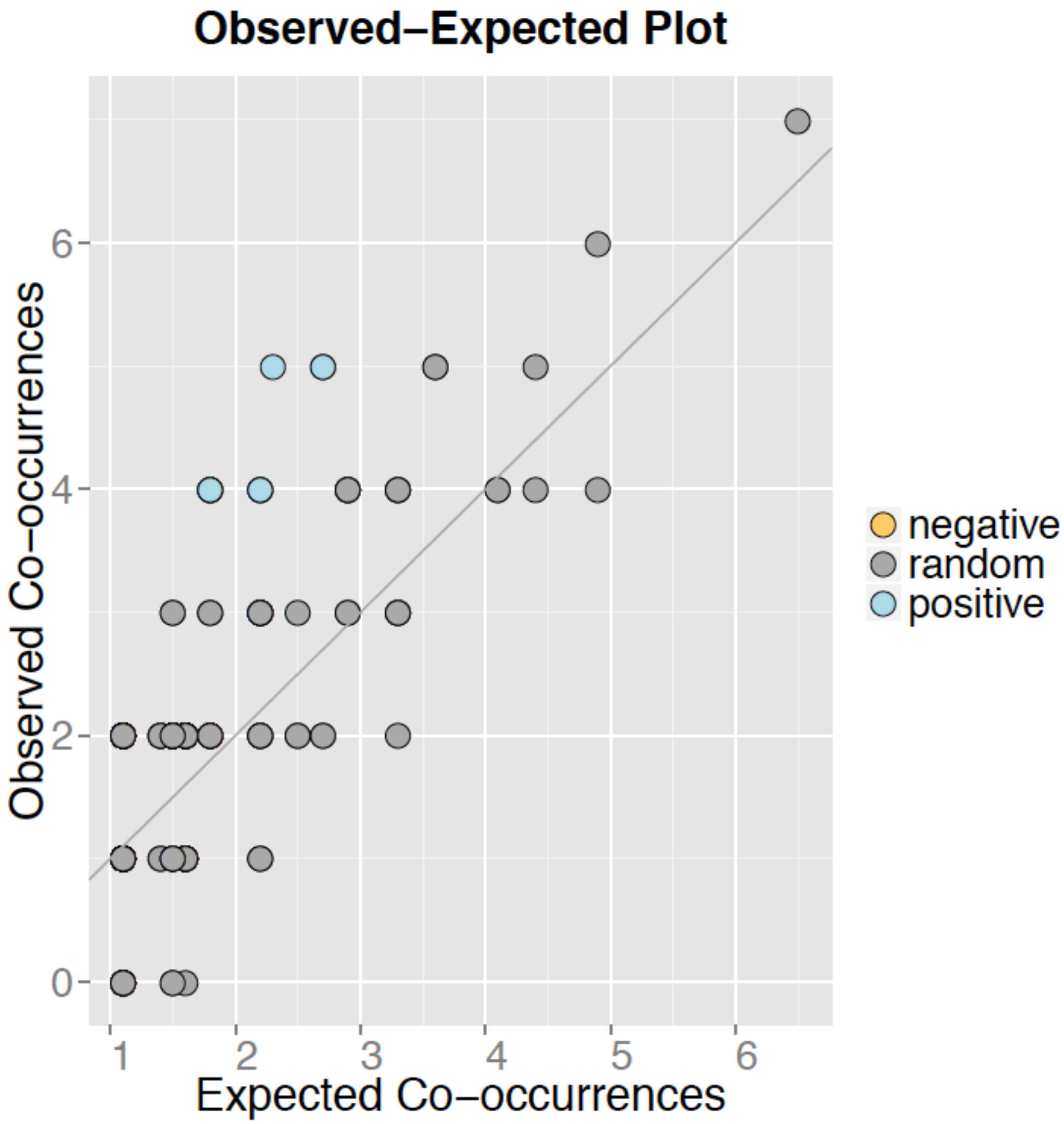

Fig. S1m) Paired OTU plot stage 72

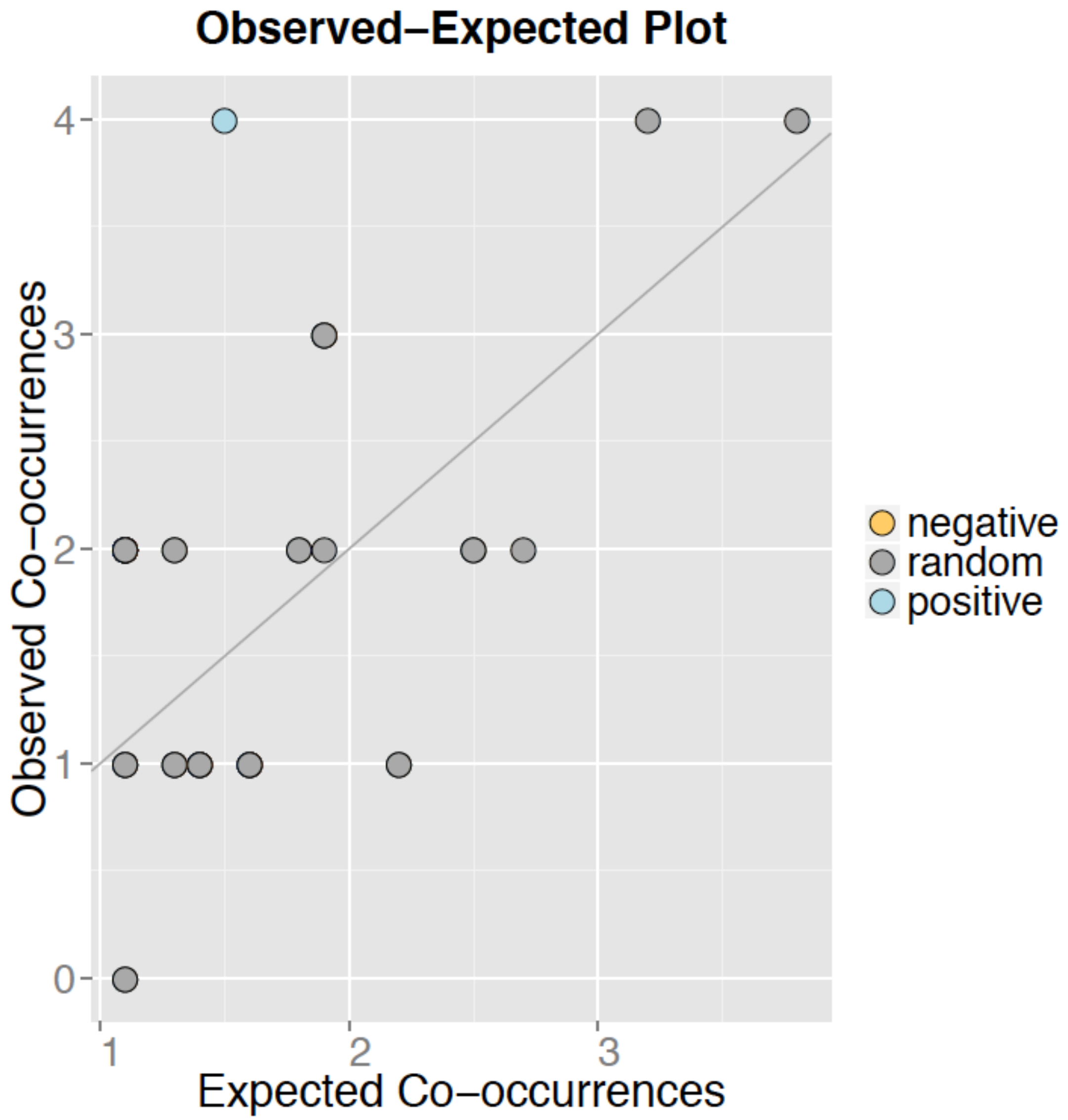

Fig. S1n) Paired OTU plot stage 80

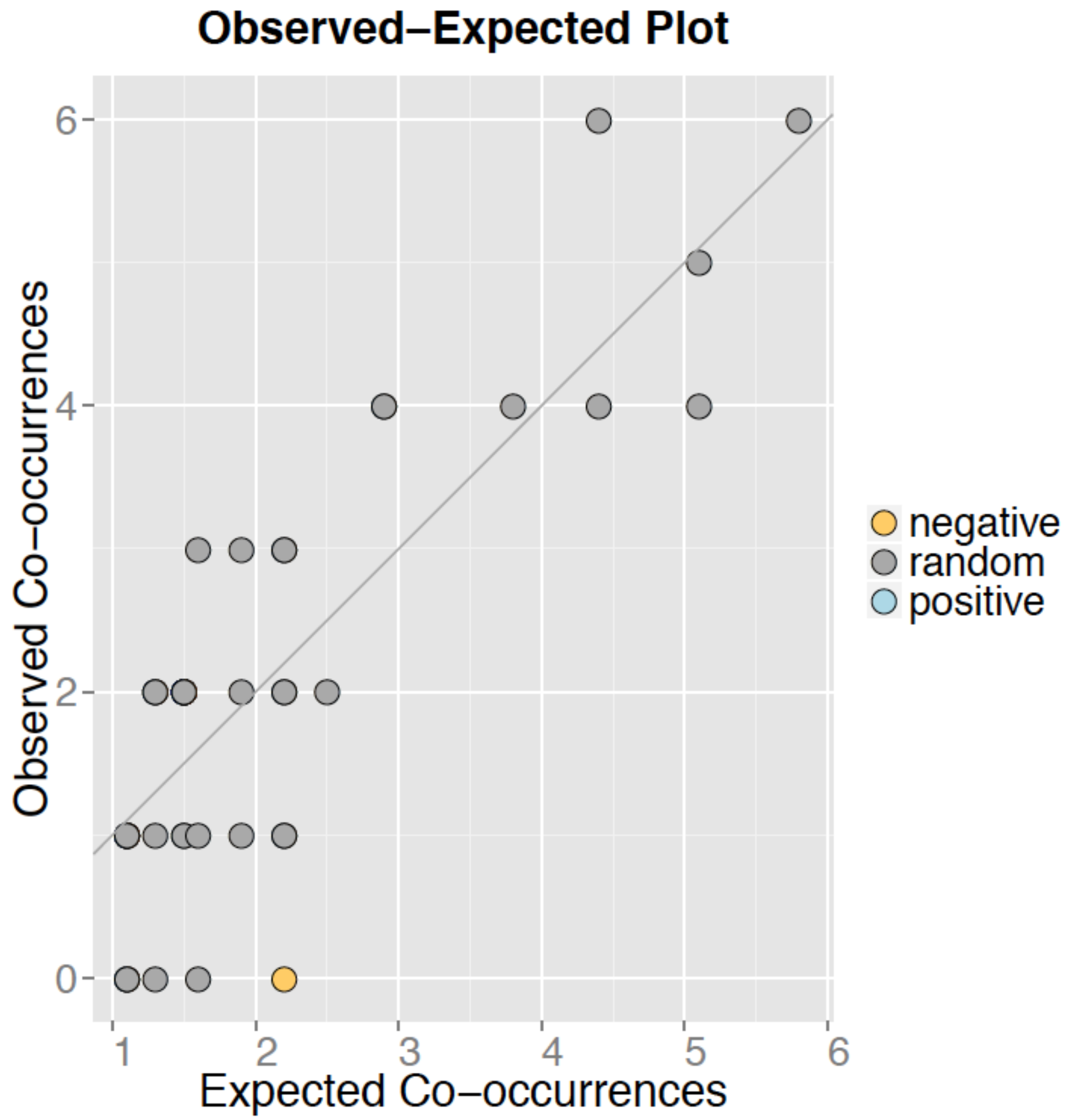

Supplement: Supplementary file 3 [file f1000research-6-14916-s0002.tgz › 7d29e7b7-86d2-4bdf-b4e1-65bd750a7be3.pdf]
