# Supplementary material for: Maize Phyllosphere Microbial Community Niche Development Across Stages of Host Leaf Growth
Source: F1000Res. 2018 Jan 18;6:1698. Originally published 2017 Sep 18. [Version 3] doi: 10.12688/f1000research.12490.3 (PMC5861518; doi:10.12688/f1000research.12490.3)

Figure S2 a-n Plots of SEM datatype co-occurrence at each growth stage. Fig. S2a) Stacked plot stage 30.

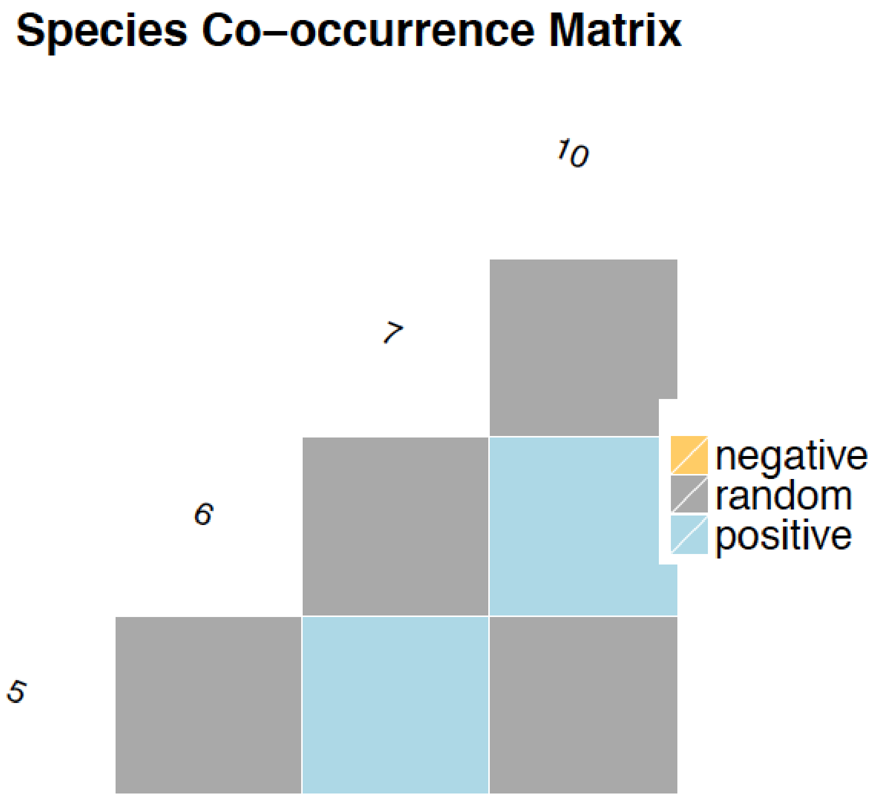

Fig. S2b) Stacked plot stage 41. (Note—no pairs were significant at this stage.)

Species Co–occurrence Matrix

Fig. S2c) Stacked plot stage 48.

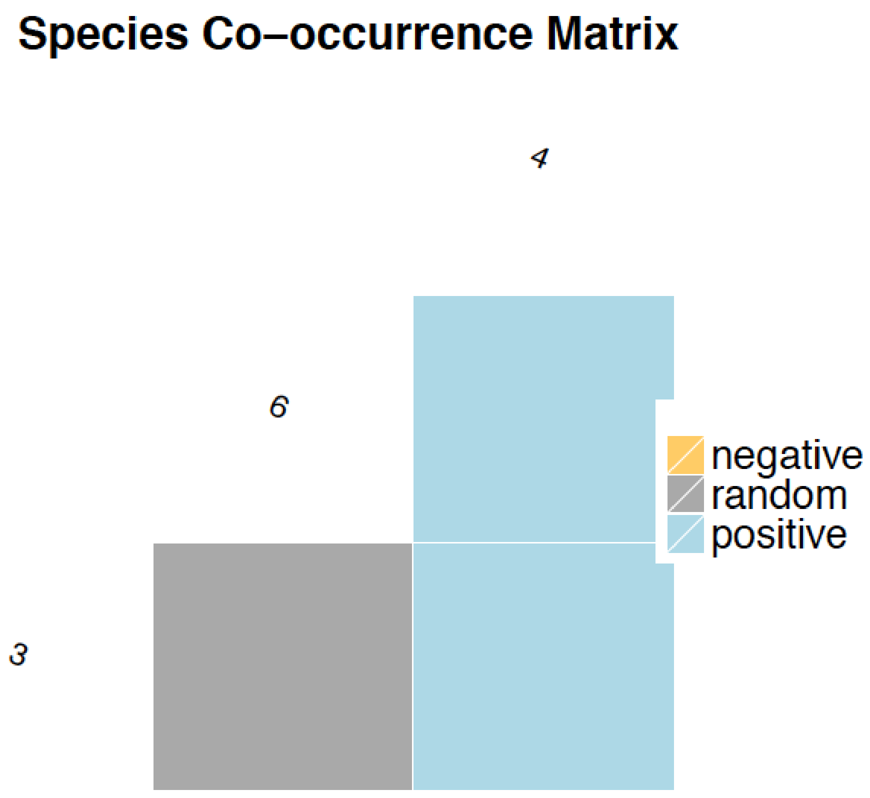

Fig. S2d) Stacked plot stage 58.

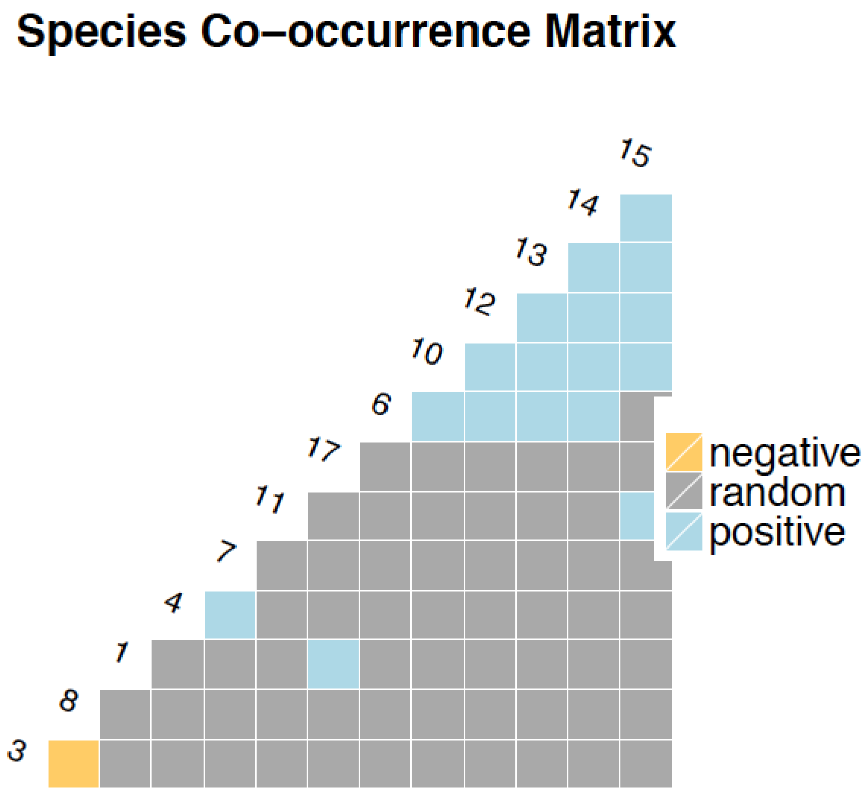

Fig. S2e) Stacked plot stage 62.

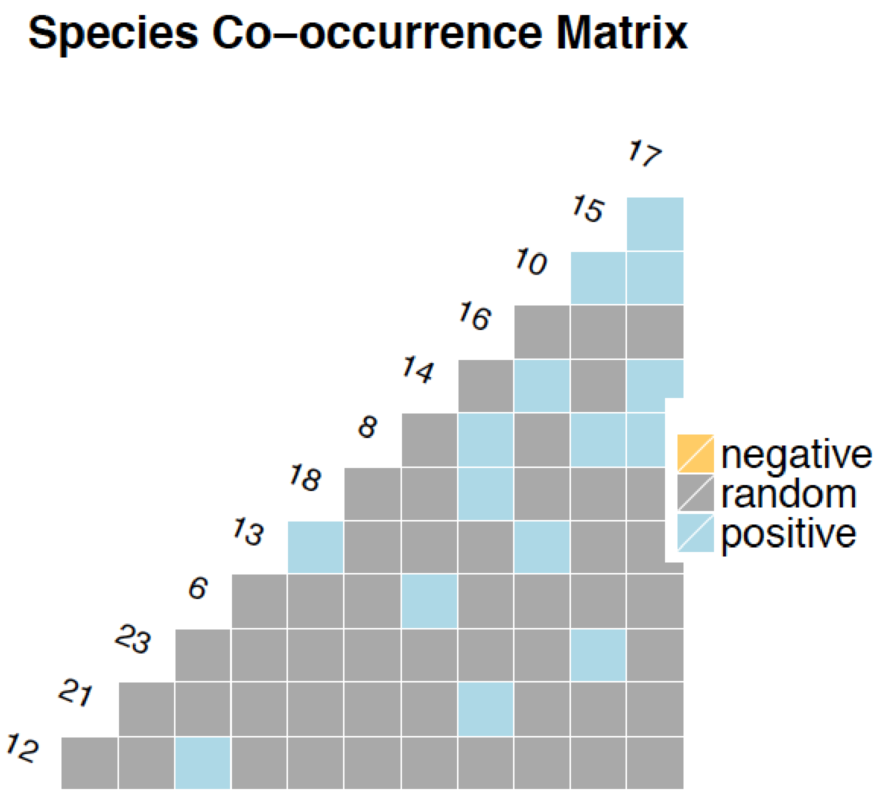

Fig. S2f) Stacked plot stage 72.

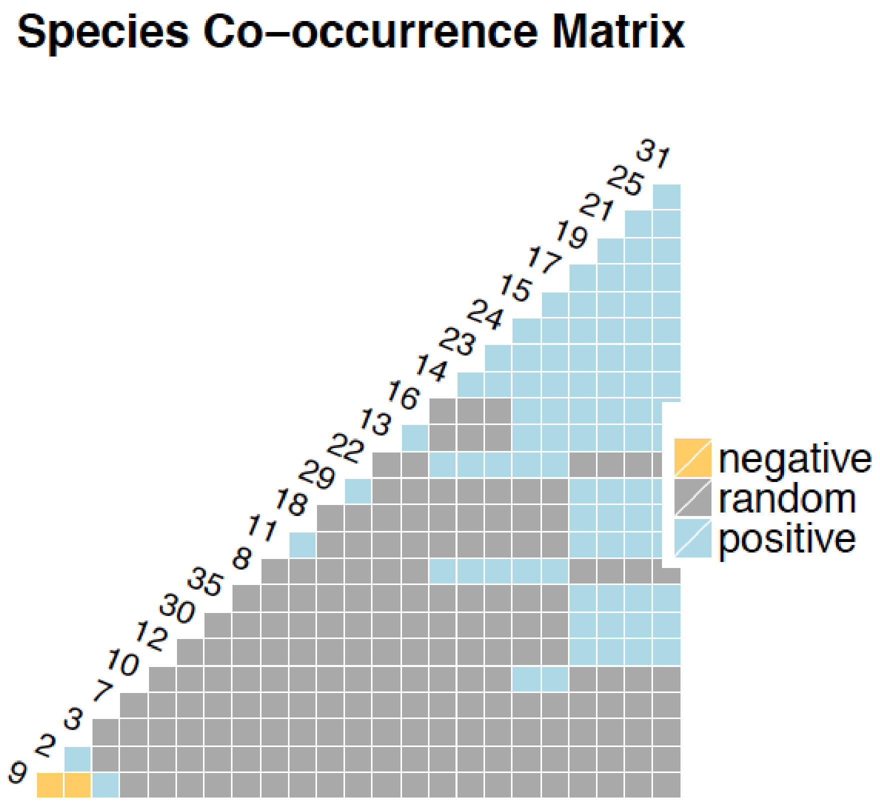

Fig. S2g) Stacked plot stage 80.

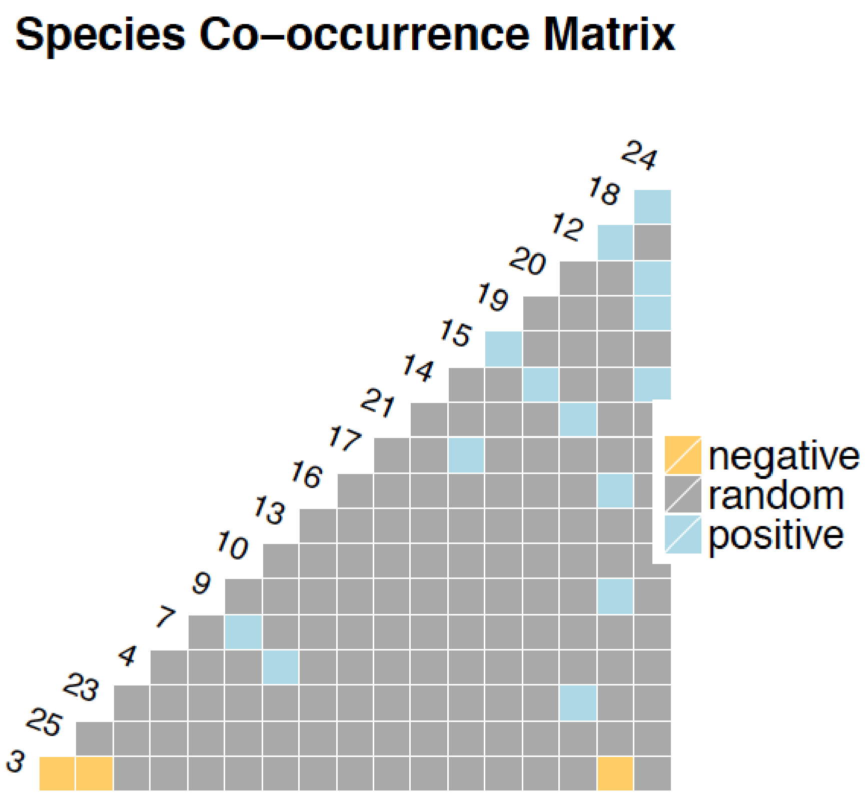

Fig. S1h) Paired OTU plot stage 30

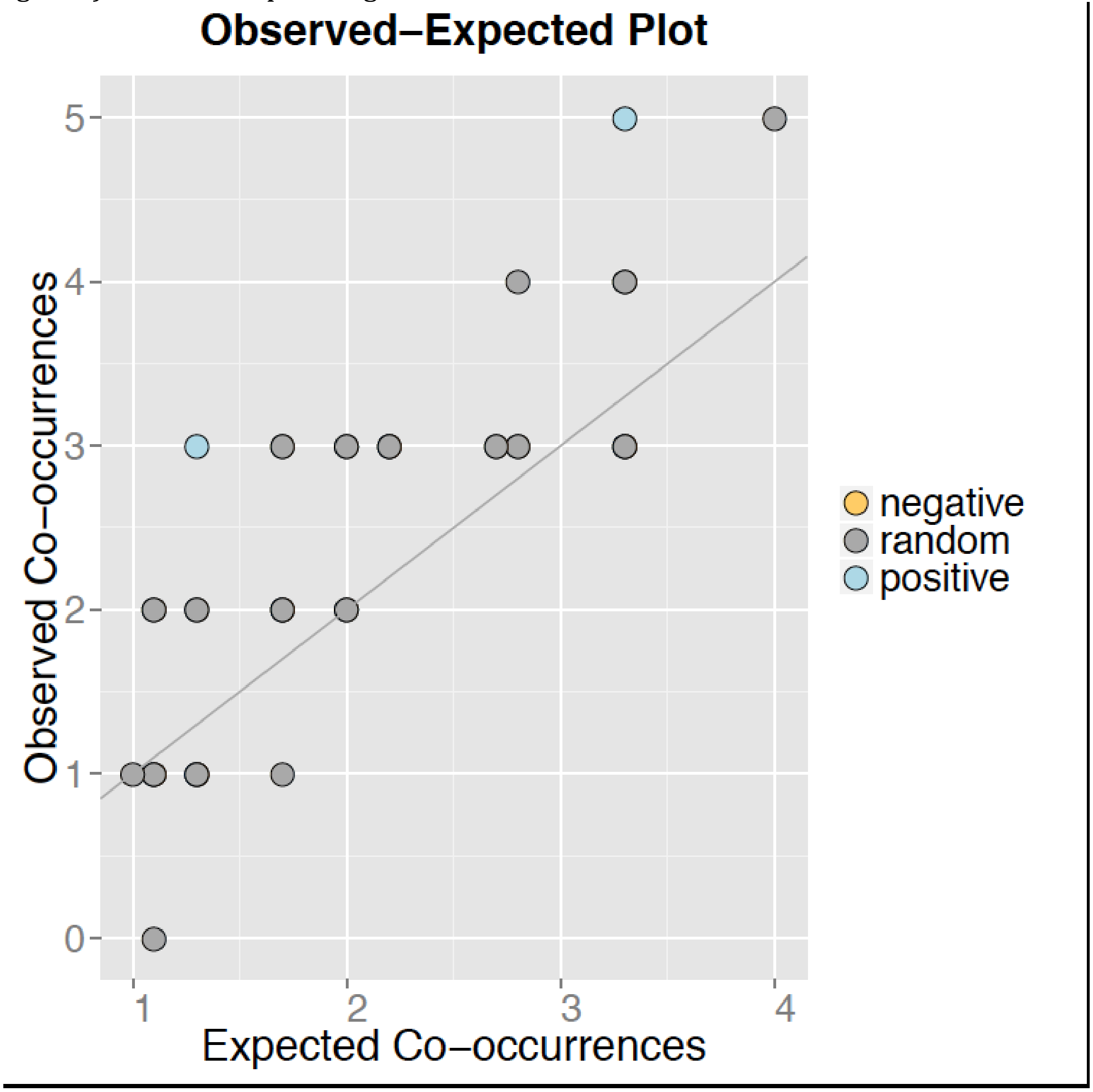

Fig. S1i) Paired OTU plot stage 41

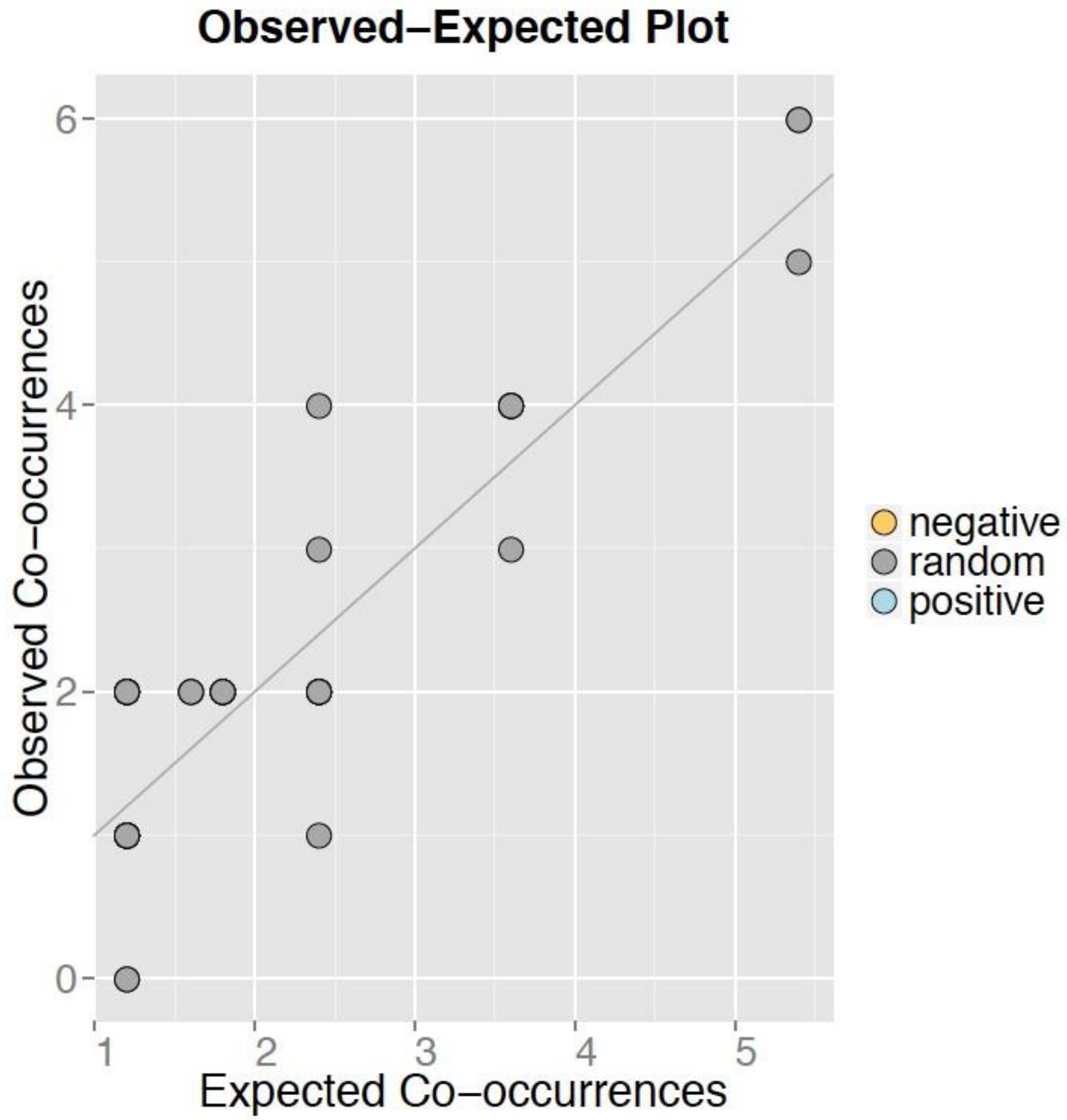

Fig. S1j) Paired OTU plot stage 48

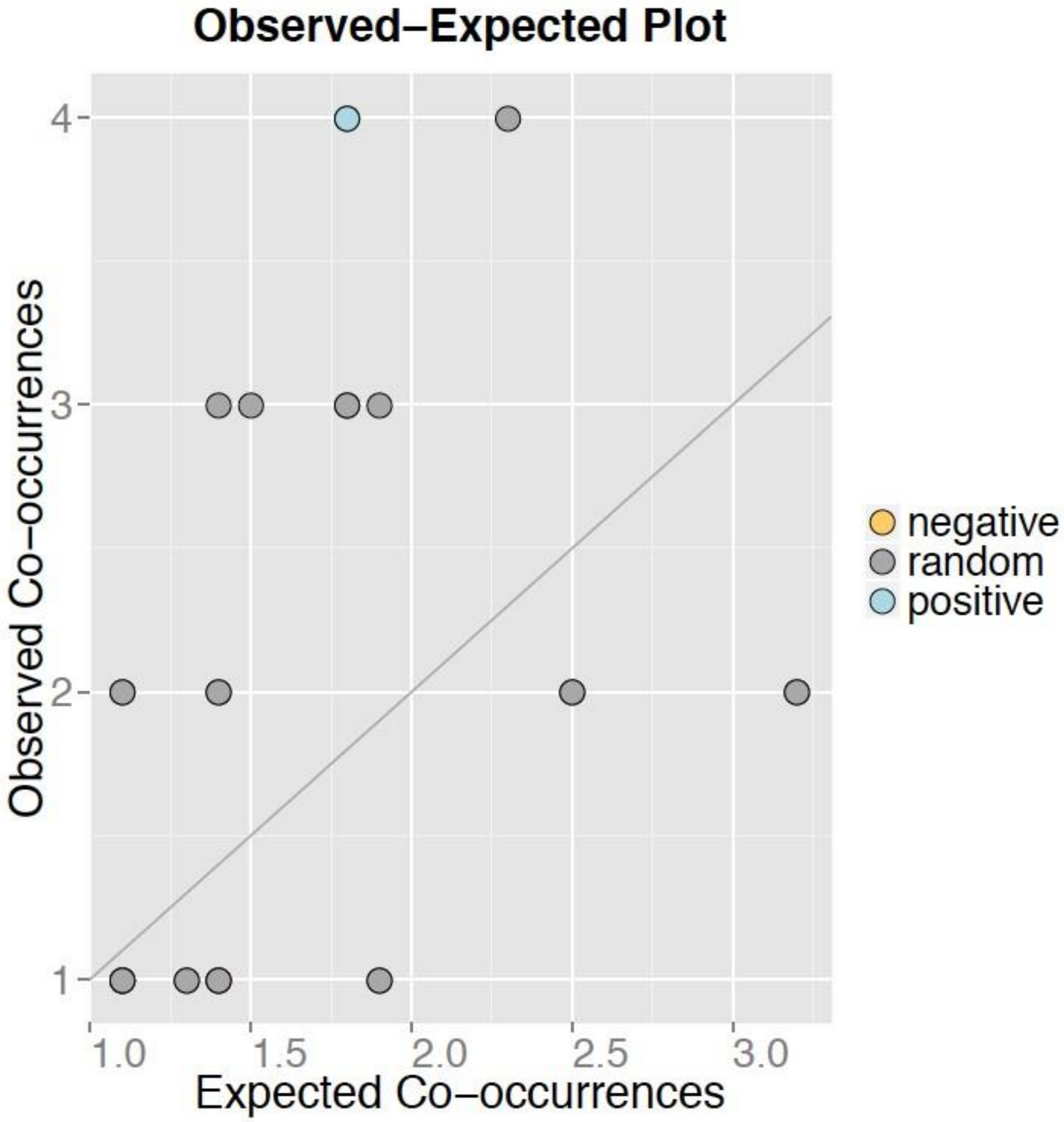

Fig. S1k) Paired OTU plot stage 58

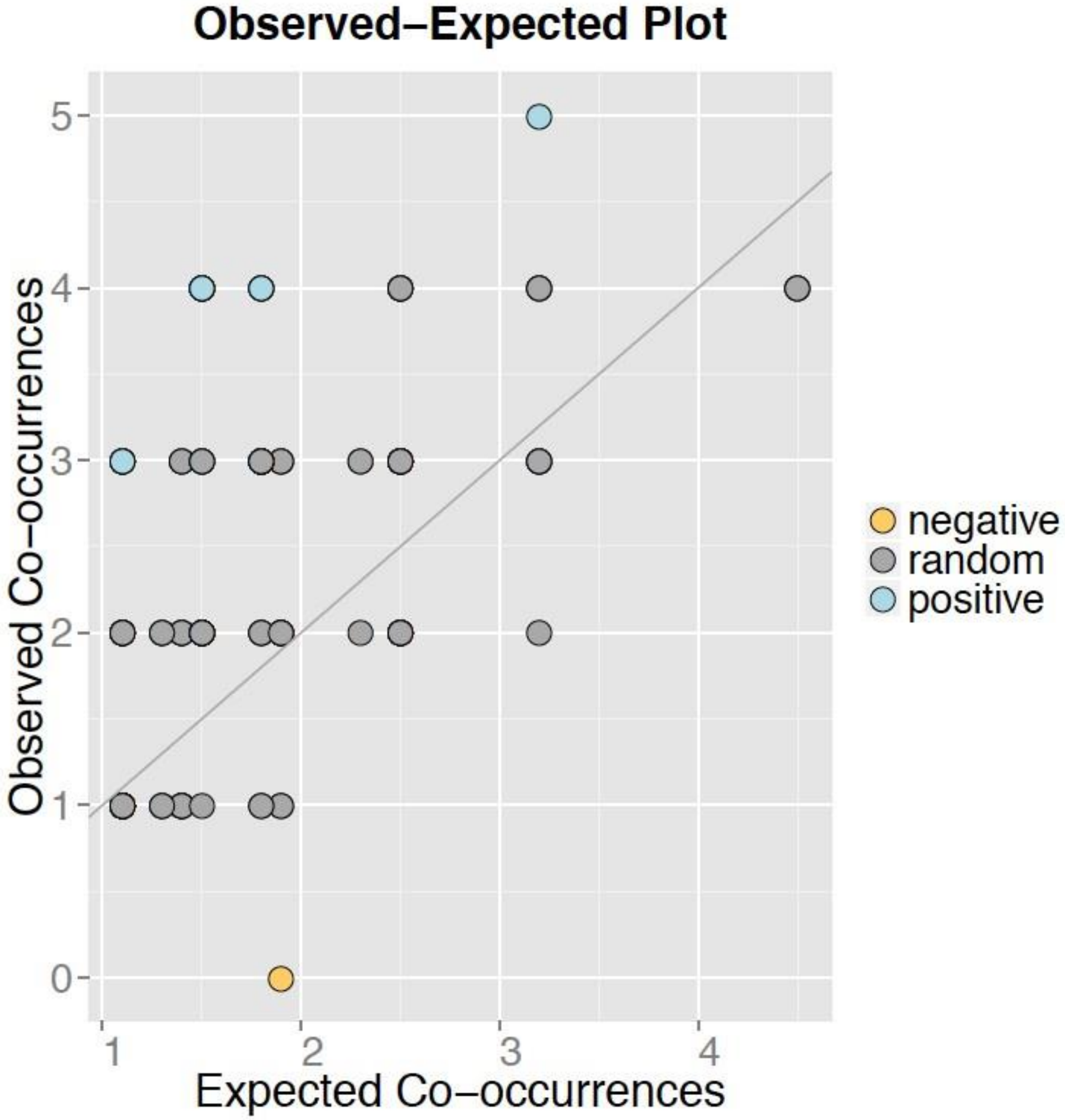

Fig. S1l) Paired OTU plot stage 62

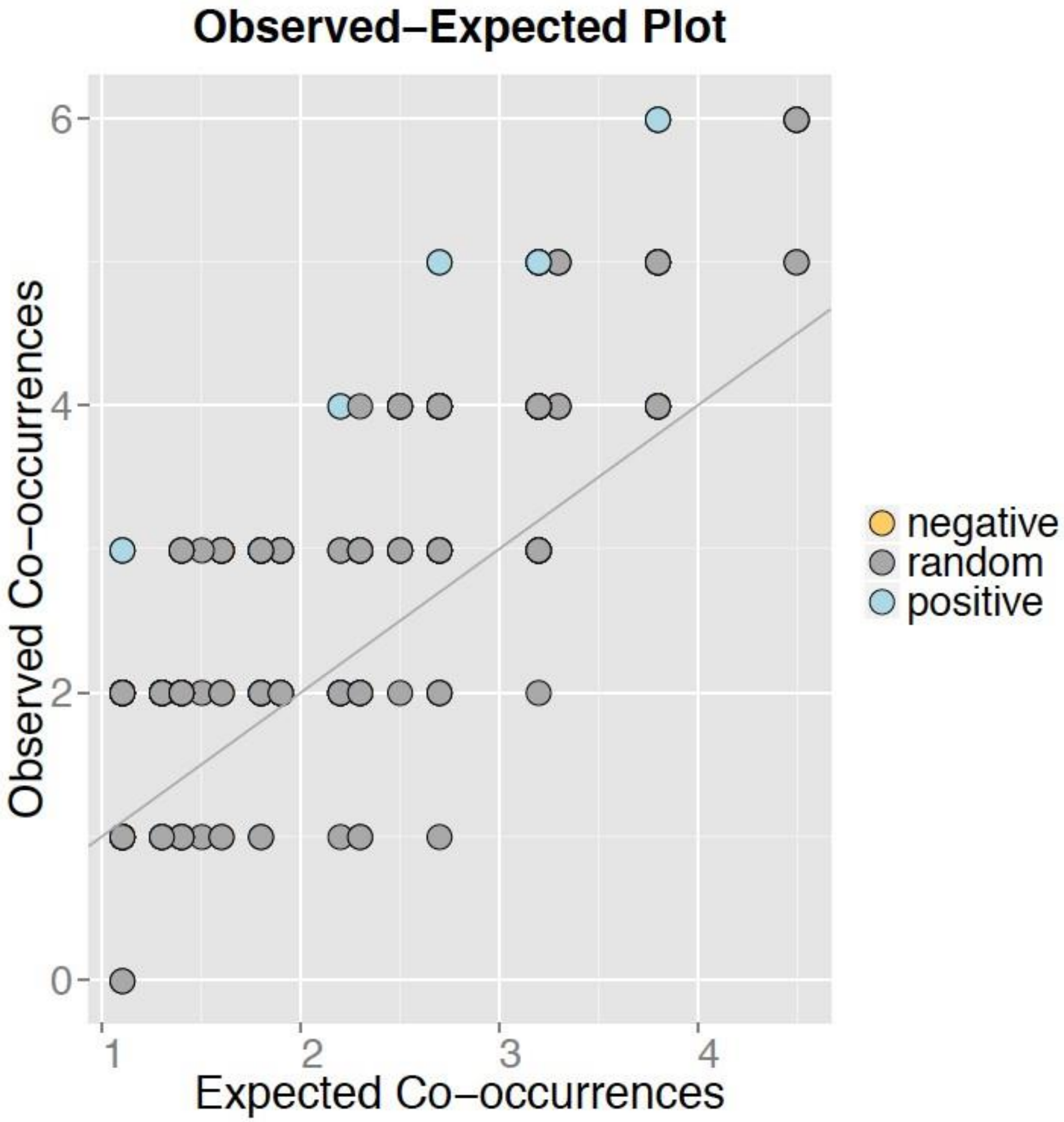

Fig. S1m) Paired OTU plot stage 72

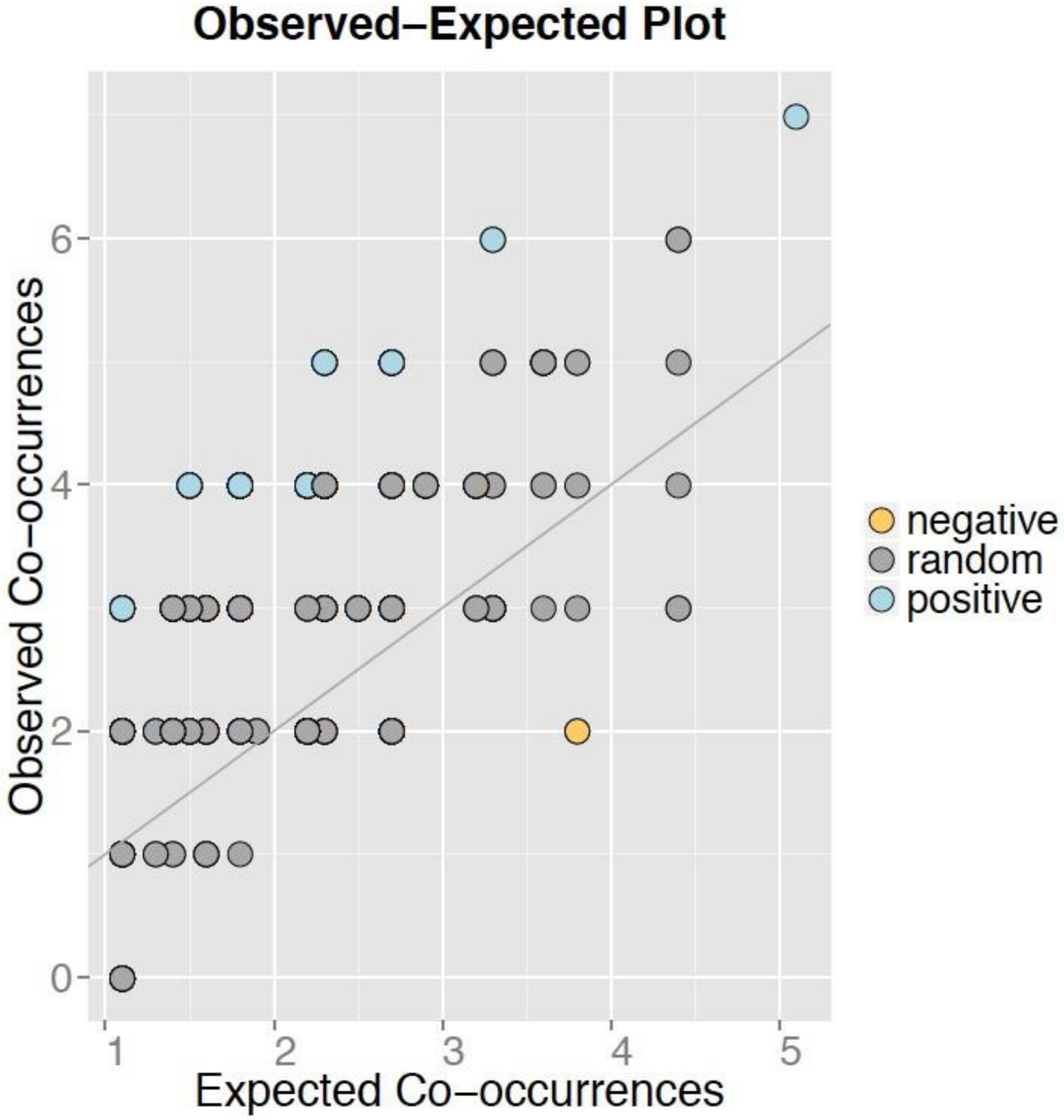

Fig. S1n) Paired OTU plot stage 80

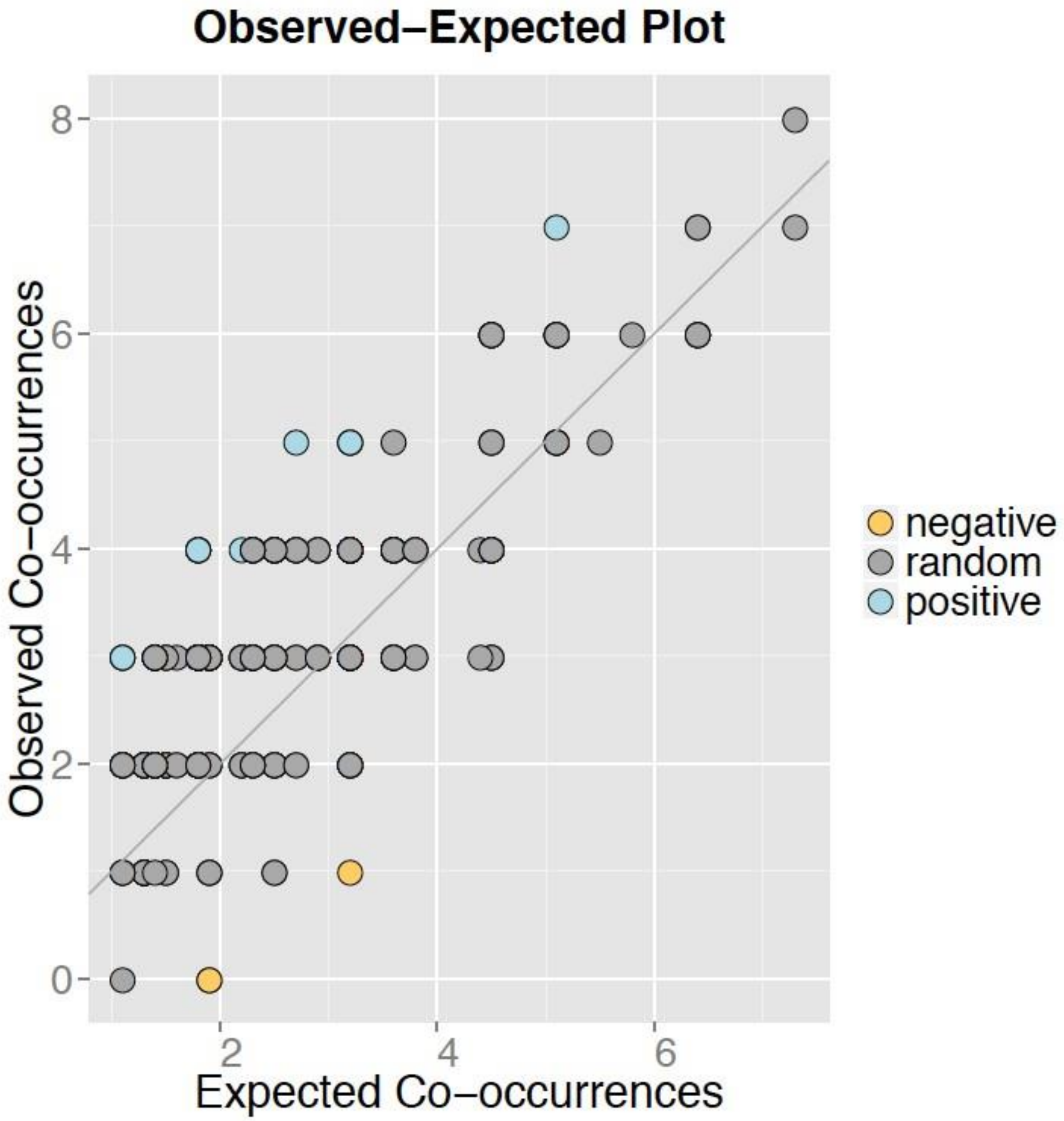

Supplement: Supplementary file 4 [file f1000research-6-14916-s0003.tgz › 5e9a6601-9c36-4032-a7eb-8e97822a97bf.pdf]
