# Supplementary material for: Maize Phyllosphere Microbial Community Niche Development Across Stages of Host Leaf Growth
Source: F1000Res. 2018 Jan 18;6:1698. Originally published 2017 Sep 18. [Version 3] doi: 10.12688/f1000research.12490.3 (PMC5861518; doi:10.12688/f1000research.12490.3)
Supplement: Supplementary file 5 [file f1000research-6-14916-s0004.tgz › 14124192-05cf-446b-b65e-f16e9ee8ac14.pdf]

SFig3 SEM Core SCU

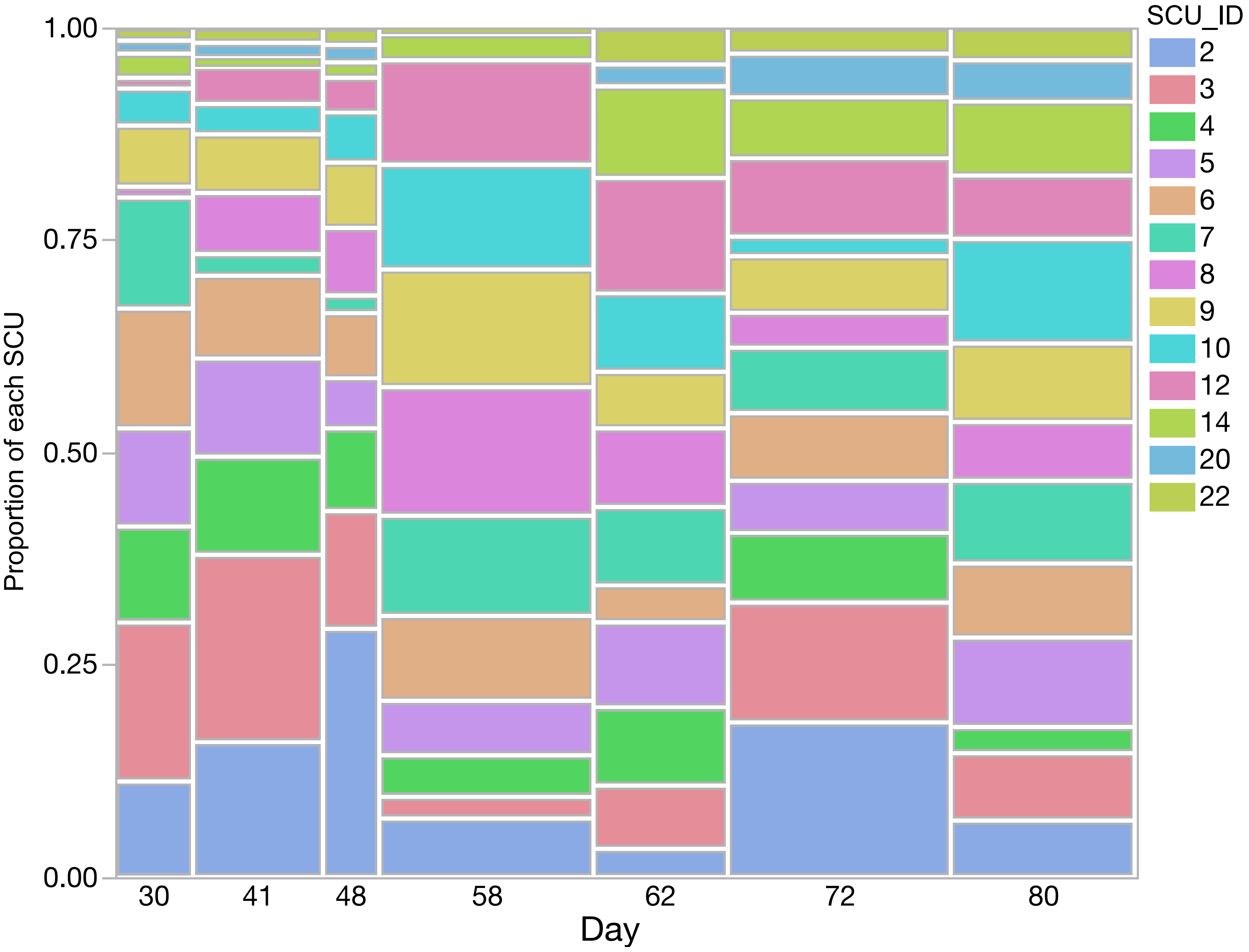

Supplemental Figure 3 Plot of Core Microbial Cell Size-Class Units The relative amount of the core subset of SCU found in all sample dates is indicated with box sizes for each sampling day, scaled to overall abundance. Width of sample day column reflects proportion of overall SCU count.
