# Supplementary material for: Maize Phyllosphere Microbial Community Niche Development Across Stages of Host Leaf Growth
Source: F1000Res. 2018 Jan 18;6:1698. Originally published 2017 Sep 18. [Version 3] doi: 10.12688/f1000research.12490.3 (PMC5861518; doi:10.12688/f1000research.12490.3)
Supplement: Supplementary file 7 [file f1000research-6-14916-s0005.tgz › 4dac822f-51fd-4876-aae0-d1529f1e163f.pdf]

PERMANOVA
Permutational MANOVA

Resemblance worksheet
Name: Resem3
Data type: Similarity
Selection: All
Transform: Square root
Resemblance: S17 Bray Curtis similarity

Sums of squares type: Type III (partial)
Fixed effects sum to zero for mixed terms
Permutation method: Unrestricted permutation of raw data
Number of permutations: 999

Factors

Name	Abbrev.	Type	Levels
time	ti	Random	7

PERMANOVA table of results

Source	df	SS	MS	Pseudo-F	P(perm)	Unique perms
ti	6	26755	4459.2	1.4527	0.026	996
Res	56	1.719E5	3069.7			
Total	62	1.9866E5				

Details of the expected mean squares (EMS) for the model

Source	EMS
ti	1*V(Res) + 8.9841*V(ti)
Res	1*V(Res)

Construction of Pseudo-F ratio(s) from mean squares

Source	Numerator	Denominator	Num.df	Den.df
ti	1*ti	1*Res	6	56

Estimates of components of variation

Source	Estimate	Sq.root
V(ti)	154.67	12.436
V(Res)	3069.7	55.405
