# Supplementary material for: Maize Phyllosphere Microbial Community Niche Development Across Stages of Host Leaf Growth
Source: F1000Res. 2018 Jan 18;6:1698. Originally published 2017 Sep 18. [Version 3] doi: 10.12688/f1000research.12490.3 (PMC5861518; doi:10.12688/f1000research.12490.3)
Supplement: Supplementary file 8 [file f1000research-6-14916-s0006.tgz › 0486b9d8-68c6-458c-8f9c-1ca49af128f0.pdf]

PERMANOVA
Permutational MANOVA

Resemblance worksheet
Name: Resem3
Data type: Similarity
Selection: All
Transform: Square root
Resemblance: S17 Bray Curtis similarity

Sums of squares type: Type III (partial)
Fixed effects sum to zero for mixed terms
Permutation method: Unrestricted permutation of raw data
Number of permutations: 999

Factors

Name	Abbrev.	Type	Levels
time	ti	Random	7

PAIR-WISE TESTS

Term 'ti'

Groups	t	P(perm)	Unique perms
1, 2	1.1845	0.171	927
1, 3	1.1398	0.233	924
1, 4	1.0832	0.252	982
1, 5	1.14	0.177	984
1, 6	1.5303	0.018	980
1, 7	1.8239	0.001	991
2, 3	0.90976	0.588	917
2, 4	0.97118	0.448	972
2, 5	1.101	0.242	991
2, 6	1.1559	0.245	989
2, 7	1.6489	0.016	983
3, 4	0.73958	0.852	975
3, 5	1.0787	0.279	985
3, 6	0.82901	0.676	984
3, 7	1.4576	0.039	985
4, 5	0.92153	0.581	995
4, 6	0.80984	0.669	995
4, 7	1.2638	0.145	991
5, 6	1.274	0.106	996
5, 7	1.4008	0.038	992
6, 7	1.4712	0.051	994

Denominators

Groups	Denominator	Den.df
1, 2	1*Res	14
1, 3	1*Res	14
1, 4	1*Res	15
1, 5	1*Res	16
1, 6	1*Res	16
1, 7	1*Res	16
2, 3	1*Res	14
2, 4	1*Res	15
2, 5	1*Res	16
2, 6	1*Res	16
2, 7	1*Res	16

3, 4	1*Res	15
3, 5	1*Res	16
3, 6	1*Res	16
3, 7	1*Res	16
4, 5	1*Res	17
4, 6	1*Res	17
4, 7	1*Res	17
5, 6	1*Res	18
5, 7	1*Res	18
6, 7	1*Res	18

Average Similarity between/within groups

	1	2	3	4	5	6	7
1	27.253						
2	21.451	21.381					
3	21.79	21.529	21.679				
4	25.036	23.88	26.254	25.632			
5	18.984	16.392	16.948	20.622	15.107		
6	19.36	20.8	25.055	26.982	17.337	25.466	
7	22.159	20.859	23.946	28.253	20.772	26.481	36.248
