# Supplementary material for: Maize Phyllosphere Microbial Community Niche Development Across Stages of Host Leaf Growth
Source: F1000Res. 2018 Jan 18;6:1698. Originally published 2017 Sep 18. [Version 3] doi: 10.12688/f1000research.12490.3 (PMC5861518; doi:10.12688/f1000research.12490.3)
Supplement: Supplementary file 9 [file f1000research-6-14916-s0007.tgz › 110200c5-0b82-4cde-861e-3d56b2ccfbe2.pdf]

PERMDISP
Distance-based test for homogeneity of multivariate dispersions

Resemblance worksheet
Name: Resem1
Data type: Similarity
Selection: All
Transform: Square root
Resemblance: S17 Bray Curtis similarity

Group factor: age
Number of permutations: 999

Number of groups: 7
Number of samples: 229

DEVIATIONS FROM CENTROID
F: 3.8034 df1: 6 df2: 222
P(perm): 0.004

PAIRWISE COMPARISONS

Groups	t	P(perm)
(30,41)	2.2518	3.7E-2
(30,48)	1.4795	0.158
(30,58)	1.2398	0.256
(30,62)	0.26932	0.791
(30,72)	6.9565E-2	0.946
(30,80)	6.1685E-2	0.946
(41,48)	3.4834	1E-3
(41,58)	3.4752	3E-3
(41,62)	2.5946	1.3E-2
(41,72)	2.3444	3.5E-2
(41,80)	2.6561	1.9E-2
(48,58)	0.4646	0.684
(48,62)	1.2946	0.207
(48,72)	1.3699	0.225
(48,80)	1.6444	0.12
(58,62)	0.99682	0.305
(58,72)	1.0955	0.322
(58,80)	1.3542	0.172
(62,72)	0.18455	0.844
(62,80)	0.24497	0.815
(72,80)	2.1199E-2	0.988

MEANS AND STANDARD ERRORS

Group	Size	Average	SE
30	28	61.789	1.3611
41	29	55.974	2.17
48	33	64.629	1.339
58	35	63.854	1.017
62	32	62.28	1.2204
72	34	61.929	1.4427
80	38	61.892	1.027
