# Supplementary material for: Maize Phyllosphere Microbial Community Niche Development Across Stages of Host Leaf Growth
Source: F1000Res. 2018 Jan 18;6:1698. Originally published 2017 Sep 18. [Version 3] doi: 10.12688/f1000research.12490.3 (PMC5861518; doi:10.12688/f1000research.12490.3)
Supplement: Supplementary file 10 [file f1000research-6-14916-s0008.tgz › 37e16077-0843-42ff-b9f3-b2cd4d2edeb2.pdf]

SIMPER
Similarity Percentages - species contributions

One-Way Analysis

Data worksheet
Name: Data3
Data type: Abundance
Sample selection: All
Variable selection: All

Parameters
Resemblance: S17 Bray Curtis similarity
Cut off for low contributions: 90.00%

Factor Groups

Sample	time
week 1	1
week 1	1
week 1	1
week 1	1
week 1	1
week 1	1
week 1	1
week 1	1
week 2	2
week 2	2
week 2	2
week 2	2
week 2	2
week 2	2
week 2	2
week 2	2
week 3	3
week 3	3
week 3	3
week 3	3
week 3	3
week 3	3
week 3	3
week 3	3
week 4	4
week 4	4
week 4	4
week 4	4
week 4	4
week 4	4
week 4	4
week 4	4
week 4	4
week 5	5
week 5	5
week 5	5
week 5	5
week 5	5
week 5	5
week 5	5
week 5	5
week 5	5
week 5	5
week 6	6

```
week 6  6
week 6  6
week 6  6
week 6  6
week 6  6
week 6  6
week 6  6
week 6  6
week 6  6
week 7  7
week 7  7
week 7  7
week 7  7
week 7  7
week 7  7
week 7  7
week 7  7
week 7  7
week 7  7
```

Group 1
Average similarity: 27.25

Species	Av.Abund	Av.Sim	Sim/SD	Contrib%	Cum.%
23	19.00	8.57	1.56	31.44	31.44
8	23.09	5.17	0.71	18.98	50.42
5	6.94	3.08	0.34	11.29	61.71
16	14.99	3.05	0.71	11.18	72.89
26	11.93	2.10	0.67	7.70	80.59
6	11.45	1.73	0.50	6.36	86.95
11	10.11	1.24	0.45	4.54	91.49

Group 2
Average similarity: 21.38

Species	Av.Abund	Av.Sim	Sim/SD	Contrib%	Cum.%
23	9.64	13.85	0.67	64.77	64.77
8	17.86	3.69	0.46	17.25	82.02
463	16.39	0.86	0.24	4.01	86.03
21	4.81	0.54	0.34	2.51	88.54
9	6.92	0.46	0.19	2.15	90.69

Group 3
Average similarity: 21.68

Species	Av.Abund	Av.Sim	Sim/SD	Contrib%	Cum.%
8	14.46	9.86	0.51	45.49	45.49
23	7.12	4.91	0.68	22.67	68.16
11	4.05	2.49	0.32	11.47	79.64
21	7.85	1.76	0.31	8.13	87.77
16	4.29	0.92	0.19	4.24	92.00

Group 4
Average similarity: 25.63

Species	Av.Abund	Av.Sim	Sim/SD	Contrib%	Cum.%
8	23.15	13.35	1.01	52.08	52.08
23	7.40	5.46	0.56	21.32	73.40
16	9.54	2.36	0.42	9.19	82.60
6	8.54	1.92	0.43	7.47	90.07

Group 5

Average similarity: 15.11

Species	Av.Abund	Av.Sim	Sim/SD	Contrib%	Cum.%
23	8.19	2.93	0.88	19.38	19.38
8	16.80	2.69	0.43	17.81	37.19
16	12.09	1.86	0.50	12.34	49.53
21	13.10	1.35	0.85	8.96	58.49
9	9.27	1.12	0.37	7.44	65.93
26	7.20	0.70	0.51	4.66	70.60
465	7.01	0.58	0.36	3.87	74.47
69	6.31	0.52	0.37	3.44	77.91
52	4.11	0.42	0.51	2.79	80.70
7	5.55	0.35	0.26	2.30	83.00
36	3.65	0.30	0.38	1.96	84.96
1	5.06	0.29	0.15	1.95	86.91
6	6.70	0.22	0.26	1.45	88.36
15	3.45	0.20	0.15	1.34	89.70
5	3.43	0.20	0.15	1.30	91.00

Group 6
Average similarity: 25.47

Species	Av.Abund	Av.Sim	Sim/SD	Contrib%	Cum.%
8	16.18	13.99	0.60	54.94	54.94
23	6.91	5.21	0.66	20.45	75.39
21	4.30	1.91	0.38	7.49	82.88
9	5.57	1.48	0.22	5.81	88.69
17	3.43	0.95	0.23	3.73	92.42

Group 7
Average similarity: 36.25

Species	Av.Abund	Av.Sim	Sim/SD	Contrib%	Cum.%
8	33.36	18.28	1.13	50.44	50.44
25	14.79	6.75	1.12	18.62	69.06
23	7.68	3.55	0.64	9.79	78.84
21	8.75	3.42	0.63	9.44	88.29
16	12.07	1.96	0.34	5.40	93.68

Groups 1 & 2
Average dissimilarity = 78.55

	Group 1	Group 2				
Species	Av.Abund	Av.Abund	Av.Diss	Diss/SD	Contrib%	Cum.%
8	23.09	17.86	8.21	0.91	10.46	10.46
5	6.94	0.00	5.38	0.58	6.85	17.31
16	14.99	5.92	4.53	1.00	5.77	23.08
463	0.00	16.39	4.53	0.57	5.77	28.85
6	11.45	4.81	3.78	0.82	4.81	33.66
26	11.93	5.38	3.56	0.94	4.54	38.19
23	19.00	9.64	3.46	0.87	4.41	42.60
11	10.11	3.98	3.34	0.84	4.25	46.85
9	0.00	6.92	2.68	0.46	3.42	50.27
21	5.37	4.81	2.61	0.68	3.32	53.59
7	3.07	2.75	2.40	0.39	3.06	56.65
464	7.30	0.00	1.92	0.45	2.44	59.10
70	3.90	4.31	1.68	0.73	2.14	61.24
461	4.28	3.08	1.57	0.53	1.99	63.23
707	5.40	0.00	1.44	0.36	1.84	65.07
53	2.97	2.35	1.07	0.86	1.36	66.44
431	3.23	0.00	1.05	0.49	1.34	67.78
467	3.66	0.00	1.01	0.52	1.28	69.06

405	1.50	0.00	0.97	0.32	1.24	70.30
404	1.43	0.00	0.92	0.32	1.18	71.47
491	0.00	2.14	0.92	0.35	1.17	72.64
37	2.22	1.22	0.82	0.63	1.04	73.68
666	1.66	1.93	0.79	0.51	1.00	74.69
709	2.38	0.00	0.78	0.35	1.00	75.69
69	1.66	1.82	0.77	0.51	0.98	76.66
706	0.00	3.75	0.70	0.37	0.89	77.56
789	0.00	3.14	0.64	0.54	0.81	78.37
36	0.92	2.18	0.61	0.66	0.77	79.14
785	0.00	2.55	0.55	0.37	0.70	79.84
713	2.04	0.00	0.55	0.36	0.70	80.54
460	0.00	2.90	0.54	0.37	0.69	81.23
712	2.02	0.00	0.54	0.36	0.69	81.92
17	0.00	2.44	0.52	0.37	0.67	82.59
655	0.00	2.09	0.45	0.37	0.57	83.16
725	1.34	0.00	0.44	0.35	0.56	83.72
30	0.00	2.15	0.43	0.56	0.55	84.28
659	1.57	0.00	0.42	0.36	0.54	84.81
729	1.39	0.00	0.37	0.36	0.48	85.29
717	0.00	1.74	0.37	0.37	0.48	85.76
745	1.12	0.00	0.37	0.35	0.47	86.23
633	1.33	0.00	0.36	0.36	0.45	86.69
704	1.06	0.00	0.35	0.35	0.44	87.13
669	1.22	0.00	0.33	0.36	0.42	87.55
813	1.21	0.00	0.32	0.36	0.41	87.96
796	0.00	1.63	0.31	0.37	0.39	88.35
22	0.00	1.42	0.31	0.37	0.39	88.74
765	1.10	0.00	0.29	0.36	0.37	89.11
794	0.88	0.00	0.29	0.35	0.37	89.48
502	1.02	0.00	0.27	0.36	0.35	89.83
739	0.00	1.26	0.27	0.37	0.35	90.18

Groups 1 & 3
Average dissimilarity = 78.21

Species	Group 1 Av.Abund	Group 3 Av.Abund	Av.Diss	Diss/SD	Contrib%	Cum.%
8	23.09	14.46	9.58	1.00	12.24	12.24
23	19.00	7.12	6.60	1.00	8.44	20.69
5	6.94	0.00	6.45	0.69	8.25	28.93
16	14.99	4.29	5.84	1.08	7.46	36.39
6	11.45	4.48	4.97	0.90	6.35	42.74
11	10.11	4.05	4.57	0.93	5.85	48.59
26	11.93	2.21	4.34	1.04	5.55	54.14
21	5.37	7.85	4.34	0.81	5.55	59.69
9	0.00	7.68	3.30	0.52	4.22	63.91
7	3.07	2.50	3.30	0.47	4.22	68.13
464	7.30	0.00	2.24	0.47	2.86	70.99
707	5.40	0.00	1.68	0.37	2.15	73.14
70	3.90	0.00	1.35	0.57	1.73	74.87
431	3.23	0.00	1.25	0.53	1.59	76.46
461	4.28	0.00	1.24	0.37	1.59	78.05
467	3.66	0.00	1.17	0.54	1.50	79.55
405	1.50	0.00	1.17	0.36	1.50	81.06
404	1.43	0.00	1.12	0.36	1.43	82.48
53	2.97	0.00	1.00	0.76	1.28	83.76
709	2.38	0.00	0.93	0.37	1.19	84.94
12	0.00	2.56	0.90	0.35	1.15	86.09
37	2.22	0.00	0.77	0.57	0.98	87.08
24	0.00	1.18	0.65	0.33	0.83	87.91
713	2.04	0.00	0.64	0.37	0.81	88.72
712	2.02	0.00	0.63	0.37	0.81	89.53
36	0.92	1.14	0.63	0.50	0.81	90.34

Groups 2 & 3
Average dissimilarity = 78.47

Species	Group 2 Av.Abund	Group 3 Av.Abund	Av.Diss	Diss/SD	Contrib%	Cum.%
8	17.86	14.46	12.66	0.83	16.13	16.13
9	6.92	7.68	7.45	0.63	9.50	25.63
463	16.39	0.00	6.35	0.59	8.09	33.72
23	9.64	7.12	6.28	0.59	8.00	41.72
21	4.81	7.85	5.72	0.70	7.28	49.00
11	3.98	4.05	4.94	0.50	6.30	55.30
16	5.92	4.29	4.72	0.55	6.01	61.32
6	4.81	4.48	3.71	0.59	4.73	66.04
7	2.75	2.50	2.61	0.40	3.33	69.37
26	5.38	2.21	2.05	0.70	2.61	71.98
491	2.14	0.00	1.45	0.37	1.84	73.82
24	1.25	1.18	1.22	0.39	1.55	75.38
12	0.00	2.56	1.09	0.34	1.39	76.77
70	4.31	0.00	1.09	0.57	1.39	78.15
36	2.18	1.14	0.92	0.59	1.17	79.32
706	3.75	0.00	0.86	0.37	1.10	80.42
789	3.14	0.00	0.80	0.55	1.02	81.44
461	3.08	0.00	0.77	0.57	0.98	82.42
785	2.55	0.00	0.69	0.37	0.88	83.30
460	2.90	0.00	0.67	0.37	0.85	84.16
17	2.44	0.00	0.66	0.37	0.85	85.00
53	2.35	0.00	0.59	0.57	0.76	85.76
655	2.09	0.00	0.57	0.37	0.72	86.48
30	2.15	0.00	0.54	0.57	0.69	87.18
666	1.93	0.00	0.53	0.37	0.67	87.85
69	1.82	0.00	0.50	0.37	0.63	88.48
717	1.74	0.00	0.47	0.37	0.60	89.08
22	1.42	0.00	0.39	0.37	0.49	89.57
796	1.63	0.00	0.38	0.37	0.48	90.05

Groups 1 & 4
Average dissimilarity = 74.96

Species	Group 1 Av.Abund	Group 4 Av.Abund	Av.Diss	Diss/SD	Contrib%	Cum.%
8	23.09	23.15	8.32	1.03	11.10	11.10
5	6.94	1.70	5.23	0.65	6.98	18.08
23	19.00	7.40	5.22	1.13	6.96	25.05
16	14.99	9.54	5.20	1.04	6.94	31.99
6	11.45	8.54	4.63	0.95	6.17	38.16
26	11.93	1.56	3.75	0.99	5.01	43.17
11	10.11	1.27	3.27	0.83	4.36	47.54
21	5.37	4.87	2.58	0.78	3.44	50.98
467	3.66	5.18	2.44	0.77	3.26	54.23
464	7.30	0.00	2.03	0.46	2.70	56.94
7	3.07	0.00	2.00	0.35	2.67	59.61
9	0.00	2.57	1.61	0.30	2.14	61.75
707	5.40	0.00	1.52	0.37	2.03	63.78
69	1.66	3.84	1.36	0.63	1.81	65.60
70	3.90	0.00	1.21	0.56	1.62	67.22
461	4.28	0.00	1.13	0.37	1.51	68.73
431	3.23	0.00	1.11	0.52	1.48	70.21
405	1.50	0.00	0.98	0.35	1.31	71.51
404	1.43	0.00	0.93	0.35	1.24	72.75
17	0.00	1.46	0.91	0.30	1.22	73.97
1	0.00	2.39	0.91	0.33	1.21	75.18
53	2.97	0.00	0.90	0.74	1.20	76.38

709	2.38	0.00	0.83	0.37	1.10	77.48
15	0.00	1.95	0.74	0.33	0.99	78.46
36	0.92	2.05	0.73	0.63	0.98	79.44
656	0.00	1.69	0.71	0.32	0.95	80.39
37	2.22	0.00	0.69	0.56	0.92	81.31
725	1.34	0.98	0.66	0.48	0.88	82.19
59	0.00	1.66	0.63	0.33	0.84	83.03
24	0.00	2.17	0.59	0.50	0.79	83.82
713	2.04	0.00	0.58	0.37	0.77	84.59
712	2.02	0.00	0.57	0.37	0.76	85.35
52	0.00	2.04	0.56	0.51	0.74	86.09
29	1.00	1.01	0.53	0.50	0.70	86.79
666	1.66	0.00	0.47	0.37	0.63	87.42
25	0.00	1.54	0.45	0.34	0.60	88.02
659	1.57	0.00	0.45	0.37	0.59	88.61
729	1.39	0.00	0.39	0.37	0.53	89.14
745	1.12	0.00	0.39	0.37	0.52	89.65
633	1.33	0.00	0.38	0.37	0.50	90.16

Groups 2 & 4

Average dissimilarity = 76.12

	Group 2	Group 4				
Species	Av.Abund	Av.Abund	Av.Diss	Diss/SD	Contrib%	Cum.%
8	17.86	23.15	11.16	0.93	14.66	14.66
463	16.39	0.99	5.72	0.59	7.51	22.17
9	6.92	2.57	5.38	0.48	7.06	29.23
16	5.92	9.54	4.78	0.76	6.28	35.51
6	4.81	8.54	3.99	0.76	5.24	40.76
23	9.64	7.40	3.83	0.83	5.03	45.79
21	4.81	4.87	3.03	0.77	3.98	49.77
467	0.00	5.18	2.44	0.57	3.21	52.98
17	2.44	1.46	2.00	0.37	2.62	55.60
11	3.98	1.27	1.95	0.59	2.56	58.16
26	5.38	1.56	1.52	0.80	2.00	60.16
69	1.82	3.84	1.49	0.60	1.96	62.12
491	2.14	0.00	1.22	0.35	1.61	63.72
1	0.00	2.39	1.12	0.32	1.48	65.20
656	0.89	1.69	1.06	0.36	1.40	66.60
70	4.31	0.00	1.00	0.56	1.31	67.91
36	2.18	2.05	0.97	0.71	1.27	69.18
15	0.00	1.95	0.92	0.32	1.20	70.38
24	1.25	2.17	0.89	0.60	1.16	71.54
706	3.75	0.00	0.80	0.37	1.05	72.59
5	0.00	1.70	0.80	0.32	1.05	73.64
30	2.15	0.82	0.79	0.58	1.04	74.69
52	0.94	2.04	0.79	0.60	1.04	75.72
59	0.00	1.66	0.78	0.32	1.02	76.74
789	3.14	0.00	0.73	0.54	0.96	77.71
22	1.42	0.90	0.73	0.47	0.95	78.66
461	3.08	0.00	0.71	0.56	0.93	79.59
7	2.75	0.00	0.68	0.37	0.90	80.48
785	2.55	0.00	0.63	0.37	0.83	81.32
460	2.90	0.00	0.62	0.37	0.81	82.13
53	2.35	0.00	0.55	0.56	0.72	82.85
655	2.09	0.00	0.52	0.37	0.68	83.53
25	0.00	1.54	0.52	0.33	0.68	84.21
666	1.93	0.00	0.48	0.37	0.63	84.84
717	1.74	0.00	0.43	0.37	0.57	85.41
45	0.00	0.82	0.38	0.32	0.50	85.91
796	1.63	0.00	0.35	0.37	0.46	86.37
29	0.00	1.01	0.34	0.33	0.44	86.82
465	0.00	1.12	0.32	0.33	0.42	87.23
739	1.26	0.00	0.32	0.37	0.42	87.65

783	1.24	0.00	0.31	0.37	0.41	88.06
37	1.22	0.00	0.31	0.37	0.40	88.46
532	0.00	0.90	0.30	0.33	0.40	88.86
766	1.22	0.00	0.30	0.37	0.40	89.26
215	0.00	0.87	0.29	0.33	0.38	89.64
508	1.36	0.00	0.29	0.37	0.38	90.02

Groups 3 & 4
Average dissimilarity = 73.75

	Group 3	Group 4				
Species	Av.Abund	Av.Abund	Av.Diss	Diss/SD	Contrib%	Cum.%
8	14.46	23.15	12.69	0.96	17.21	17.21
16	4.29	9.54	6.71	0.83	9.10	26.31
9	7.68	2.57	6.48	0.59	8.78	35.09
23	7.12	7.40	6.11	0.79	8.29	43.38
21	7.85	4.87	5.75	0.81	7.79	51.17
6	4.48	8.54	5.67	0.84	7.69	58.86
11	4.05	1.27	4.21	0.53	5.71	64.58
467	0.00	5.18	2.93	0.65	3.97	68.54
7	2.50	0.00	1.99	0.34	2.69	71.24
17	0.00	1.46	1.75	0.33	2.38	73.61
24	1.18	2.17	1.54	0.53	2.09	75.71
26	2.21	1.56	1.40	0.48	1.90	77.61
69	0.00	3.84	1.40	0.52	1.90	79.50
1	0.00	2.39	1.35	0.34	1.84	81.34
12	2.56	0.00	1.14	0.36	1.54	82.88
36	1.14	2.05	1.12	0.62	1.51	84.39
656	0.00	1.69	1.11	0.34	1.50	85.90
15	0.00	1.95	1.10	0.34	1.50	87.39
5	0.00	1.70	0.96	0.34	1.30	88.70
59	0.00	1.66	0.94	0.34	1.27	89.97
52	0.00	2.04	0.75	0.52	1.01	90.98

Groups 1 & 5
Average dissimilarity = 81.02

	Group 1	Group 5				
Species	Av.Abund	Av.Abund	Av.Diss	Diss/SD	Contrib%	Cum.%
8	23.09	16.80	6.06	0.95	7.48	7.48
16	14.99	12.09	4.18	0.71	5.16	12.64
5	6.94	3.43	3.20	0.60	3.95	16.59
23	19.00	8.19	3.16	0.79	3.90	20.49
6	11.45	6.70	2.99	0.84	3.69	24.18
26	11.93	7.20	2.79	0.96	3.45	27.63
9	0.00	9.27	2.68	0.67	3.30	30.93
21	5.37	13.10	2.56	0.83	3.16	34.09
11	10.11	1.99	2.42	0.80	2.99	37.08
7	3.07	5.55	2.12	0.52	2.62	39.70
464	7.30	2.52	1.87	0.55	2.31	42.01
467	3.66	4.68	1.81	0.54	2.24	44.24
465	0.00	7.01	1.68	0.60	2.07	46.32
1	0.00	5.06	1.51	0.47	1.87	48.18
69	1.66	6.31	1.42	0.75	1.76	49.94
707	5.40	0.00	1.15	0.35	1.42	51.36
15	0.00	3.45	1.03	0.47	1.27	52.63
22	0.00	7.02	1.01	0.33	1.25	53.88
25	0.00	1.59	0.92	0.29	1.14	55.02
501	0.00	2.91	0.90	0.35	1.11	56.13
70	3.90	0.00	0.90	0.53	1.11	57.24
59	0.00	2.98	0.89	0.47	1.10	58.34
461	4.28	0.00	0.87	0.35	1.07	59.41
36	0.92	3.65	0.82	0.78	1.01	60.42

431	3.23	0.00	0.81	0.49	0.99	61.41
52	0.00	4.11	0.79	0.83	0.98	62.39
702	0.00	5.31	0.75	0.46	0.93	63.32
53	2.97	0.00	0.67	0.69	0.83	64.14
405	1.50	0.00	0.62	0.32	0.76	64.91
10	0.00	2.94	0.60	0.47	0.74	65.65
709	2.38	0.00	0.60	0.35	0.74	66.39
404	1.43	0.00	0.59	0.32	0.73	67.12
17	0.00	2.43	0.58	0.60	0.72	67.84
37	2.22	0.92	0.58	0.59	0.71	68.55
745	1.12	2.27	0.56	0.58	0.69	69.24
12	0.00	3.75	0.54	0.33	0.67	69.91
725	1.34	1.70	0.51	0.51	0.63	70.53
30	0.00	2.49	0.48	0.56	0.59	71.13
24	0.00	1.58	0.47	0.47	0.58	71.71
31	0.00	2.46	0.47	0.48	0.58	72.28
29	1.00	0.77	0.44	0.45	0.55	72.83
801	0.00	3.41	0.44	0.44	0.54	73.37
713	2.04	0.00	0.44	0.35	0.54	73.91
27	0.00	3.01	0.43	0.33	0.54	74.45
712	2.02	0.00	0.43	0.35	0.53	74.98
265	0.00	1.11	0.40	0.31	0.49	75.48
771	0.00	2.78	0.40	0.43	0.49	75.97
708	0.94	1.04	0.39	0.48	0.48	76.45
765	1.10	0.89	0.38	0.47	0.47	76.92
731	0.95	0.97	0.38	0.48	0.46	77.39
796	0.00	1.76	0.37	0.32	0.46	77.85
19	0.00	2.55	0.37	0.33	0.45	78.30
817	0.00	2.83	0.37	0.47	0.45	78.75
794	0.88	1.08	0.36	0.48	0.45	79.20
760	0.00	1.69	0.36	0.32	0.44	79.64
666	1.66	0.00	0.35	0.35	0.44	80.08
659	1.57	0.00	0.34	0.35	0.41	80.49
724	0.00	1.45	0.31	0.32	0.38	80.87
810	0.00	2.29	0.30	0.48	0.38	81.24
815	0.00	2.45	0.30	0.33	0.37	81.61
729	1.39	0.00	0.30	0.35	0.37	81.98
654	0.00	0.96	0.29	0.32	0.36	82.34
633	1.33	0.00	0.28	0.35	0.35	82.69
266	0.00	0.78	0.28	0.31	0.35	83.04
762	0.00	2.22	0.27	0.33	0.33	83.37
704	1.06	0.00	0.27	0.35	0.33	83.70
669	1.22	0.00	0.26	0.35	0.32	84.02
813	1.21	0.00	0.26	0.35	0.32	84.34
693	0.00	1.44	0.25	0.48	0.31	84.66
791	0.00	1.66	0.25	0.33	0.31	84.97
783	0.00	1.59	0.24	0.33	0.30	85.27
758	0.00	1.60	0.23	0.33	0.29	85.56
811	0.00	1.50	0.23	0.33	0.28	85.84
785	0.00	1.49	0.23	0.33	0.28	86.12
658	0.00	0.73	0.22	0.32	0.27	86.39
502	1.02	0.00	0.22	0.35	0.27	86.66
670	0.00	1.51	0.22	0.33	0.27	86.93
565	0.00	0.72	0.22	0.32	0.27	87.20
45	0.00	0.73	0.22	0.32	0.27	87.47
728	0.00	1.63	0.21	0.49	0.27	87.73
735	0.00	1.02	0.21	0.32	0.27	88.00
683	0.94	0.00	0.20	0.35	0.25	88.24
763	0.00	1.30	0.20	0.33	0.24	88.49
552	0.91	0.00	0.19	0.35	0.24	88.73
68	0.00	1.32	0.19	0.33	0.23	88.96
617	0.00	0.90	0.19	0.32	0.23	89.20
690	0.88	0.00	0.19	0.35	0.23	89.43
823	0.88	0.00	0.19	0.35	0.23	89.66

761	0.00	1.23	0.19	0.33	0.23	89.90
614	0.00	0.88	0.18	0.32	0.23	90.12

Groups 2 & 5
Average dissimilarity = 83.61

Species	Group 2 Av.Abund	Group 5 Av.Abund	Av.Diss	Diss/SD	Contrib%	Cum.%
8	17.86	16.80	7.20	0.69	8.62	8.62
16	5.92	12.09	4.84	0.50	5.79	14.41
9	6.92	9.27	4.23	0.67	5.06	19.47
463	16.39	0.00	4.00	0.54	4.78	24.25
21	4.81	13.10	2.85	0.88	3.41	27.66
465	0.00	7.01	1.96	0.55	2.34	30.00
26	5.38	7.20	1.81	0.92	2.16	32.16
23	9.64	8.19	1.77	0.49	2.12	34.27
1	0.00	5.06	1.76	0.46	2.10	36.37
7	2.75	5.55	1.68	0.63	2.01	38.39
69	1.82	6.31	1.58	0.70	1.89	40.28
467	0.00	4.68	1.57	0.36	1.88	42.16
6	4.81	6.70	1.56	0.81	1.86	44.02
25	0.00	1.59	1.41	0.27	1.69	45.71
11	3.98	1.99	1.35	0.61	1.62	47.33
22	1.42	7.02	1.31	0.40	1.56	48.89
15	0.00	3.45	1.20	0.46	1.43	50.32
5	0.00	3.43	1.19	0.46	1.42	51.74
501	0.00	2.91	1.10	0.33	1.31	53.05
59	0.00	2.98	1.03	0.46	1.24	54.29
17	2.44	2.43	1.03	0.68	1.23	55.52
36	2.18	3.65	1.00	0.79	1.19	56.71
491	2.14	0.77	0.93	0.38	1.11	57.83
52	0.94	4.11	0.93	0.79	1.11	58.94
702	0.00	5.31	0.79	0.45	0.95	59.88
70	4.31	0.00	0.78	0.54	0.93	60.82
706	3.75	1.25	0.76	0.43	0.91	61.73
30	2.15	2.49	0.75	0.69	0.90	62.63
24	1.25	1.58	0.71	0.56	0.85	63.47
785	2.55	1.49	0.69	0.47	0.82	64.29
10	0.00	2.94	0.67	0.46	0.81	65.10
796	1.63	1.76	0.63	0.46	0.76	65.86
460	2.90	1.02	0.60	0.44	0.71	66.57
464	0.00	2.52	0.59	0.46	0.70	67.28
789	3.14	0.00	0.57	0.52	0.68	67.96
12	0.00	3.75	0.57	0.32	0.68	68.64
771	0.90	2.78	0.55	0.54	0.66	69.30
461	3.08	0.00	0.55	0.54	0.66	69.96
31	0.00	2.46	0.51	0.47	0.62	70.58
724	1.13	1.45	0.49	0.45	0.59	71.17
265	0.00	1.11	0.49	0.30	0.58	71.75
783	1.24	1.59	0.47	0.48	0.56	72.31
801	0.00	3.41	0.46	0.43	0.55	72.86
27	0.00	3.01	0.46	0.32	0.55	73.41
53	2.35	0.00	0.43	0.54	0.51	73.91
791	0.95	1.66	0.41	0.46	0.49	74.40
655	2.09	0.00	0.40	0.36	0.48	74.88
760	0.00	1.69	0.39	0.32	0.47	75.35
19	0.00	2.55	0.39	0.32	0.46	75.81
817	0.00	2.83	0.38	0.47	0.46	76.27
666	1.93	0.00	0.37	0.36	0.44	76.71
728	1.02	1.63	0.36	0.60	0.43	77.14
766	1.22	1.16	0.36	0.48	0.43	77.57
745	0.00	2.27	0.36	0.49	0.42	78.00
739	1.26	0.97	0.34	0.46	0.41	78.41
266	0.00	0.78	0.34	0.30	0.41	78.82

804	0.96	1.39	0.34	0.49	0.41	79.22
29	0.00	0.77	0.34	0.30	0.41	79.63
727	1.01	0.75	0.34	0.47	0.40	80.03
654	0.00	0.96	0.34	0.31	0.40	80.44
717	1.74	0.00	0.33	0.36	0.40	80.84
37	1.22	0.92	0.33	0.46	0.39	81.23
810	0.00	2.29	0.32	0.48	0.38	81.61
656	0.89	0.81	0.31	0.46	0.37	81.99
815	0.00	2.45	0.31	0.33	0.37	82.36
653	0.94	0.80	0.29	0.48	0.34	82.70
762	0.00	2.22	0.28	0.33	0.34	83.04
693	0.00	1.44	0.27	0.47	0.33	83.37
658	0.00	0.73	0.26	0.31	0.31	83.67
565	0.00	0.72	0.25	0.31	0.30	83.97
45	0.00	0.73	0.25	0.31	0.30	84.27
758	0.00	1.60	0.24	0.32	0.29	84.57
811	0.00	1.50	0.24	0.32	0.29	84.86
708	0.00	1.04	0.24	0.32	0.29	85.14
735	0.00	1.02	0.24	0.32	0.28	85.43
725	0.00	1.70	0.23	0.49	0.28	85.71
508	1.36	0.00	0.23	0.36	0.28	85.98
670	0.00	1.51	0.23	0.32	0.27	86.26
731	0.00	0.97	0.22	0.32	0.27	86.53
733	1.25	0.00	0.21	0.36	0.25	86.78
763	0.00	1.30	0.21	0.32	0.25	87.03
355	1.08	0.00	0.21	0.36	0.25	87.28
617	0.00	0.90	0.21	0.32	0.25	87.53
819	1.08	0.00	0.21	0.36	0.25	87.77
765	0.00	0.89	0.20	0.32	0.24	88.02
764	1.05	0.00	0.20	0.35	0.24	88.26
614	0.00	0.88	0.20	0.32	0.24	88.50
68	0.00	1.32	0.20	0.32	0.24	88.74
761	0.00	1.23	0.20	0.32	0.24	88.98
830	1.03	0.00	0.20	0.36	0.24	89.22
457	1.02	0.00	0.20	0.35	0.24	89.45
640	0.00	0.84	0.19	0.32	0.23	89.68
488	1.14	0.00	0.19	0.36	0.23	89.91
800	0.99	0.00	0.19	0.36	0.23	90.14

Groups 3 & 5
Average dissimilarity = 83.05

Species	Group 3 Av.Abund	Group 5 Av.Abund	Av.Diss	Diss/SD	Contrib%	Cum.%
8	14.46	16.80	8.11	0.80	9.76	9.76
16	4.29	12.09	5.74	0.62	6.91	16.67
9	7.68	9.27	5.13	0.77	6.18	22.85
21	7.85	13.10	4.53	0.86	5.45	28.30
23	7.12	8.19	2.93	0.69	3.52	31.82
6	4.48	6.70	2.59	0.64	3.12	34.94
7	2.50	5.55	2.40	0.56	2.89	37.82
11	4.05	1.99	2.34	0.55	2.82	40.64
465	0.00	7.01	2.30	0.59	2.77	43.41
1	0.00	5.06	2.08	0.49	2.51	45.92
26	2.21	7.20	2.06	0.84	2.48	48.40
467	0.00	4.68	1.87	0.39	2.25	50.66
69	0.00	6.31	1.68	0.69	2.02	52.68
25	0.00	1.59	1.68	0.31	2.02	54.70
15	0.00	3.45	1.42	0.49	1.71	56.41
5	0.00	3.43	1.41	0.49	1.70	58.11
12	2.56	3.75	1.35	0.48	1.62	59.73
501	0.00	2.91	1.31	0.36	1.58	61.31
59	0.00	2.98	1.23	0.49	1.48	62.79
22	0.00	7.02	1.19	0.33	1.43	64.22

36	1.14	3.65	1.15	0.76	1.38	65.60
24	1.18	1.58	1.05	0.52	1.27	66.86
52	0.00	4.11	1.02	0.80	1.23	68.09
702	0.00	5.31	0.88	0.46	1.06	69.15
10	0.00	2.94	0.78	0.48	0.94	70.09
17	0.00	2.43	0.78	0.61	0.93	71.02
464	0.00	2.52	0.68	0.48	0.82	71.84
30	0.00	2.49	0.61	0.55	0.74	72.58
31	0.00	2.46	0.59	0.49	0.71	73.30
265	0.00	1.11	0.59	0.33	0.71	74.00
27	0.00	3.01	0.51	0.33	0.61	74.62
801	0.00	3.41	0.51	0.44	0.61	75.23
771	0.00	2.78	0.47	0.43	0.56	75.79
796	0.00	1.76	0.47	0.33	0.56	76.35
760	0.00	1.69	0.45	0.33	0.54	76.89
19	0.00	2.55	0.43	0.33	0.52	77.41
817	0.00	2.83	0.42	0.48	0.51	77.92
266	0.00	0.78	0.41	0.33	0.50	78.42
29	0.00	0.77	0.41	0.33	0.49	78.91
654	0.00	0.96	0.40	0.33	0.48	79.39
745	0.00	2.27	0.40	0.50	0.48	79.87
724	0.00	1.45	0.38	0.33	0.46	80.33
810	0.00	2.29	0.35	0.49	0.43	80.76
815	0.00	2.45	0.34	0.33	0.41	81.17
693	0.00	1.44	0.31	0.48	0.38	81.55
762	0.00	2.22	0.31	0.33	0.37	81.92
658	0.00	0.73	0.30	0.33	0.37	82.29
791	0.00	1.66	0.30	0.33	0.36	82.65
565	0.00	0.72	0.30	0.33	0.36	83.01
45	0.00	0.73	0.30	0.33	0.36	83.37
783	0.00	1.59	0.29	0.33	0.35	83.71
708	0.00	1.04	0.28	0.33	0.33	84.05
811	0.00	1.50	0.27	0.33	0.33	84.37
758	0.00	1.60	0.27	0.33	0.33	84.70
735	0.00	1.02	0.27	0.33	0.33	85.03
785	0.00	1.49	0.27	0.33	0.32	85.35
725	0.00	1.70	0.26	0.50	0.31	85.66
731	0.00	0.97	0.26	0.33	0.31	85.98
670	0.00	1.51	0.26	0.33	0.31	86.28
728	0.00	1.63	0.25	0.50	0.30	86.58
617	0.00	0.90	0.24	0.33	0.29	86.87
765	0.00	0.89	0.24	0.33	0.28	87.16
763	0.00	1.30	0.23	0.33	0.28	87.44
614	0.00	0.88	0.23	0.33	0.28	87.72
68	0.00	1.32	0.22	0.33	0.27	87.99
761	0.00	1.23	0.22	0.33	0.27	88.25
640	0.00	0.84	0.22	0.33	0.27	88.52
721	0.00	0.82	0.22	0.33	0.26	88.79
656	0.00	0.81	0.22	0.33	0.26	89.05
491	0.00	0.77	0.21	0.33	0.25	89.29
808	0.00	0.77	0.20	0.33	0.25	89.54
786	0.00	1.44	0.20	0.33	0.24	89.78
701	0.00	0.75	0.20	0.33	0.24	90.02

Groups 4 & 5
Average dissimilarity = 79.38

	Group 4	Group 5				
Species	Av.Abund	Av.Abund	Av.Diss	Diss/SD	Contrib%	Cum.%
8	23.15	16.80	7.21	0.84	9.09	9.09
16	9.54	12.09	5.07	0.61	6.38	15.47
9	2.57	9.27	3.95	0.65	4.98	20.44
21	4.87	13.10	2.99	0.91	3.76	24.21
6	8.54	6.70	2.97	0.81	3.74	27.95

467	5.18	4.68	2.74	0.62	3.46	31.41
23	7.40	8.19	2.63	0.76	3.31	34.72
1	2.39	5.06	2.33	0.56	2.93	37.65
465	1.12	7.01	2.13	0.62	2.68	40.33
69	3.84	6.31	1.95	0.79	2.46	42.79
26	1.56	7.20	1.69	0.88	2.13	44.92
15	1.95	3.45	1.66	0.55	2.09	47.00
25	1.54	1.59	1.63	0.35	2.05	49.05
5	1.70	3.43	1.59	0.56	2.01	51.06
7	0.00	5.55	1.45	0.57	1.83	52.89
59	1.66	2.98	1.42	0.55	1.79	54.68
22	0.90	7.02	1.39	0.41	1.75	56.43
17	1.46	2.43	1.31	0.46	1.65	58.07
501	0.00	2.91	1.14	0.35	1.44	59.51
52	2.04	4.11	1.11	0.86	1.40	60.91
36	2.05	3.65	1.11	0.82	1.39	62.30
24	2.17	1.58	0.93	0.67	1.17	63.47
11	1.27	1.99	0.90	0.54	1.13	64.60
702	0.00	5.31	0.83	0.46	1.04	65.64
656	1.69	0.81	0.77	0.38	0.97	66.62
30	0.82	2.49	0.73	0.61	0.92	67.53
10	0.00	2.94	0.71	0.47	0.89	68.43
464	0.00	2.52	0.62	0.48	0.78	69.21
12	0.00	3.75	0.60	0.33	0.75	69.96
29	1.01	0.77	0.56	0.44	0.70	70.66
31	0.00	2.46	0.54	0.49	0.68	71.34
265	0.00	1.11	0.51	0.32	0.64	71.98
45	0.82	0.73	0.48	0.44	0.61	72.59
801	0.00	3.41	0.48	0.44	0.61	73.20
27	0.00	3.01	0.48	0.33	0.60	73.80
771	0.00	2.78	0.44	0.43	0.56	74.36
796	0.00	1.76	0.43	0.33	0.54	74.89
725	0.98	1.70	0.42	0.55	0.53	75.43
760	0.00	1.69	0.41	0.33	0.52	75.94
19	0.00	2.55	0.41	0.33	0.51	76.45
817	0.00	2.83	0.40	0.48	0.50	76.96
745	0.00	2.27	0.37	0.49	0.47	77.43
266	0.00	0.78	0.36	0.32	0.45	77.88
654	0.00	0.96	0.35	0.32	0.45	78.32
724	0.00	1.45	0.35	0.33	0.44	78.77
810	0.00	2.29	0.33	0.48	0.42	79.19
815	0.00	2.45	0.33	0.33	0.41	79.60
762	0.00	2.22	0.29	0.33	0.37	79.97
693	0.00	1.44	0.29	0.48	0.36	80.33
791	0.00	1.66	0.28	0.33	0.36	80.69
78	0.79	0.72	0.28	0.43	0.35	81.04
783	0.00	1.59	0.27	0.33	0.34	81.38
658	0.00	0.73	0.27	0.32	0.34	81.72
565	0.00	0.72	0.26	0.32	0.33	82.05
758	0.00	1.60	0.26	0.33	0.32	82.38
811	0.00	1.50	0.26	0.33	0.32	82.70
708	0.00	1.04	0.25	0.33	0.32	83.02
785	0.00	1.49	0.25	0.33	0.32	83.34
735	0.00	1.02	0.25	0.33	0.31	83.65
670	0.00	1.51	0.24	0.33	0.30	83.95
731	0.00	0.97	0.24	0.33	0.30	84.25
728	0.00	1.63	0.24	0.49	0.30	84.55
532	0.90	0.00	0.23	0.32	0.29	84.84
463	0.99	0.00	0.22	0.33	0.28	85.12
742	0.99	0.00	0.22	0.33	0.28	85.40
215	0.87	0.00	0.22	0.32	0.28	85.68
763	0.00	1.30	0.22	0.33	0.28	85.96
617	0.00	0.90	0.22	0.33	0.28	86.23
28	0.97	0.00	0.22	0.33	0.27	86.51

765	0.00	0.89	0.22	0.33	0.27	86.78
614	0.00	0.88	0.21	0.33	0.27	87.05
219	0.83	0.00	0.21	0.32	0.27	87.32
538	0.83	0.00	0.21	0.32	0.27	87.58
68	0.00	1.32	0.21	0.33	0.26	87.85
761	0.00	1.23	0.21	0.33	0.26	88.11
640	0.00	0.84	0.20	0.33	0.26	88.37
721	0.00	0.82	0.20	0.33	0.25	88.62
798	0.89	0.00	0.20	0.33	0.25	88.87
786	0.00	1.44	0.19	0.33	0.24	89.11
491	0.00	0.77	0.19	0.33	0.24	89.35
808	0.00	0.77	0.19	0.33	0.24	89.58
804	0.00	1.39	0.19	0.33	0.23	89.82
744	0.82	0.00	0.18	0.33	0.23	90.05

Groups 1 & 6
Average dissimilarity = 80.64

	Group 1	Group 6				
Species	Av.Abund	Av.Abund	Av.Diss	Diss/SD	Contrib%	Cum.%
8	23.09	16.18	9.69	1.06	12.01	12.01
23	19.00	6.91	6.09	1.05	7.55	19.56
5	6.94	2.30	5.85	0.75	7.25	26.82
16	14.99	0.00	5.17	1.11	6.41	33.23
6	11.45	0.00	4.17	0.84	5.17	38.40
26	11.93	0.00	4.09	1.01	5.07	43.47
11	10.11	2.29	3.80	0.89	4.71	48.18
21	5.37	4.30	2.94	0.86	3.65	51.83
9	0.00	5.57	2.91	0.51	3.61	55.44
7	3.07	1.13	2.65	0.44	3.29	58.73
467	3.66	4.58	2.62	0.76	3.25	61.98
464	7.30	0.00	2.20	0.47	2.73	64.70
17	0.00	3.43	1.77	0.52	2.20	66.90
707	5.40	0.00	1.65	0.37	2.05	68.95
70	3.90	0.00	1.33	0.57	1.65	70.60
461	4.28	0.00	1.23	0.37	1.52	72.12
431	3.23	0.00	1.22	0.53	1.51	73.63
405	1.50	0.00	1.11	0.37	1.38	75.00
59	0.00	2.41	1.10	0.44	1.36	76.37
404	1.43	0.00	1.05	0.37	1.31	77.67
15	0.00	2.33	1.02	0.45	1.27	78.94
53	2.97	0.00	0.98	0.76	1.21	80.15
709	2.38	0.00	0.91	0.37	1.12	81.28
362	0.00	1.99	0.82	0.31	1.02	82.30
37	2.22	0.00	0.76	0.57	0.94	83.24
25	0.00	1.44	0.67	0.30	0.84	84.07
713	2.04	0.00	0.63	0.37	0.78	84.85
712	2.02	0.00	0.62	0.37	0.77	85.62
30	0.00	1.14	0.61	0.30	0.76	86.38
24	0.00	1.12	0.61	0.30	0.75	87.13
363	0.00	1.25	0.52	0.31	0.64	87.78
69	1.66	0.00	0.51	0.37	0.63	88.41
725	1.34	0.00	0.51	0.37	0.63	89.04
666	1.66	0.00	0.51	0.37	0.63	89.68
468	0.00	1.30	0.50	0.31	0.62	90.30

Groups 2 & 6
Average dissimilarity = 79.20

	Group 2	Group 6				
Species	Av.Abund	Av.Abund	Av.Diss	Diss/SD	Contrib%	Cum.%
8	17.86	16.18	14.07	0.80	17.76	17.76
9	6.92	5.57	6.64	0.62	8.39	26.15

463	16.39	0.00	6.16	0.59	7.78	33.93
23	9.64	6.91	4.74	0.70	5.98	39.91
21	4.81	4.30	3.82	0.76	4.82	44.73
17	2.44	3.43	3.01	0.55	3.80	48.52
11	3.98	2.29	2.66	0.62	3.36	51.88
467	0.00	4.58	2.63	0.54	3.32	55.20
7	2.75	1.13	1.50	0.45	1.89	57.09
59	0.00	2.41	1.49	0.40	1.88	58.96
16	5.92	0.00	1.48	0.57	1.86	60.83
491	2.14	0.00	1.38	0.37	1.74	62.57
26	5.38	0.00	1.37	0.76	1.73	64.30
5	0.00	2.30	1.37	0.42	1.73	66.03
15	0.00	2.33	1.36	0.43	1.72	67.75
30	2.15	1.14	1.32	0.41	1.67	69.43
6	4.81	0.00	1.20	0.57	1.51	70.94
24	1.25	1.12	1.15	0.36	1.45	72.39
70	4.31	0.00	1.07	0.57	1.35	73.75
362	0.00	1.99	1.05	0.29	1.33	75.08
25	0.00	1.44	0.91	0.29	1.15	76.23
706	3.75	0.00	0.85	0.37	1.08	77.31
789	3.14	0.00	0.79	0.55	1.00	78.30
461	3.08	0.00	0.76	0.57	0.96	79.26
785	2.55	0.00	0.68	0.37	0.86	80.12
363	0.00	1.25	0.66	0.29	0.84	80.96
460	2.90	0.00	0.66	0.37	0.84	81.80
10	0.00	0.85	0.63	0.28	0.79	82.59
468	0.00	1.30	0.62	0.30	0.79	83.38
53	2.35	0.00	0.59	0.57	0.74	84.11
465	0.00	1.07	0.57	0.29	0.71	84.83
655	2.09	0.00	0.56	0.37	0.71	85.54
36	2.18	0.00	0.54	0.57	0.68	86.21
409	0.00	1.00	0.53	0.29	0.67	86.88
666	1.93	0.00	0.52	0.37	0.65	87.54
69	1.82	0.00	0.49	0.37	0.62	88.15
717	1.74	0.00	0.47	0.37	0.59	88.74
675	0.00	0.87	0.46	0.29	0.58	89.33
22	1.42	0.00	0.38	0.37	0.48	89.81
796	1.63	0.00	0.37	0.37	0.47	90.28

Groups 3 & 6
Average dissimilarity = 74.94

	Group 3	Group 6				
Species	Av.Abund	Av.Abund	Av.Diss	Diss/SD	Contrib%	Cum.%
8	14.46	16.18	15.26	0.98	20.37	20.37
9	7.68	5.57	8.22	0.77	10.96	31.33
21	7.85	4.30	6.66	0.92	8.89	40.22
23	7.12	6.91	5.73	1.01	7.64	47.87
11	4.05	2.29	4.82	0.71	6.43	54.30
16	4.29	0.00	3.97	0.55	5.30	59.60
6	4.48	0.00	3.44	0.55	4.60	64.20
467	0.00	4.58	3.17	0.62	4.23	68.42
17	0.00	3.43	3.08	0.55	4.12	72.54
7	2.50	1.13	3.01	0.47	4.02	76.56
24	1.18	1.12	1.91	0.48	2.55	79.11
59	0.00	2.41	1.79	0.46	2.39	81.50
5	0.00	2.30	1.65	0.48	2.20	83.70
15	0.00	2.33	1.64	0.48	2.19	85.89
362	0.00	1.99	1.27	0.32	1.70	87.59
12	2.56	0.00	1.27	0.37	1.69	89.28
25	0.00	1.44	1.10	0.32	1.47	90.75

Groups 4 & 6

Average dissimilarity = 73.02

Species	Group 4 Av.Abund	Group 6 Av.Abund	Av.Diss	Diss/SD	Contrib%	Cum.%
8	23.15	16.18	12.42	0.91	17.02	17.02
9	2.57	5.57	5.65	0.58	7.74	24.76
23	7.40	6.91	5.19	0.92	7.11	31.87
16	9.54	0.00	5.02	0.82	6.88	38.74
467	5.18	4.58	4.27	0.86	5.84	44.58
6	8.54	0.00	4.26	0.80	5.84	50.42
21	4.87	4.30	3.85	0.88	5.27	55.70
17	1.46	3.43	3.34	0.59	4.57	60.27
15	1.95	2.33	2.21	0.58	3.02	63.29
59	1.66	2.41	2.14	0.56	2.94	66.23
5	1.70	2.30	2.07	0.57	2.84	69.06
11	1.27	2.29	1.91	0.53	2.61	71.68
24	2.17	1.12	1.49	0.49	2.04	73.71
25	1.54	1.44	1.41	0.43	1.93	75.64
69	3.84	0.00	1.37	0.53	1.88	77.52
1	2.39	0.00	1.30	0.35	1.79	79.30
30	0.82	1.14	1.24	0.41	1.69	81.00
362	0.00	1.99	1.08	0.31	1.48	82.48
656	1.69	0.00	1.06	0.35	1.45	83.93
465	1.12	1.07	0.88	0.44	1.21	85.14
7	0.00	1.13	0.83	0.30	1.13	86.27
36	2.05	0.00	0.73	0.53	1.00	87.28
52	2.04	0.00	0.73	0.53	1.00	88.28
363	0.00	1.25	0.68	0.31	0.93	89.21
468	0.00	1.30	0.65	0.32	0.88	90.10

Groups 5 & 6

Average dissimilarity = 82.66

Species	Group 5 Av.Abund	Group 6 Av.Abund	Av.Diss	Diss/SD	Contrib%	Cum.%
8	16.80	16.18	7.81	0.90	9.45	9.45
16	12.09	0.00	5.21	0.58	6.30	15.74
9	9.27	5.57	4.68	0.75	5.66	21.40
21	13.10	4.30	3.41	0.92	4.12	25.52
467	4.68	4.58	2.97	0.61	3.59	29.11
23	8.19	6.91	2.64	0.73	3.20	32.31
465	7.01	1.07	2.40	0.64	2.90	35.21
1	5.06	0.00	2.03	0.49	2.46	37.67
15	3.45	2.33	1.95	0.64	2.35	40.03
5	3.43	2.30	1.94	0.64	2.34	42.37
25	1.59	1.44	1.90	0.39	2.29	44.66
7	5.55	1.13	1.89	0.64	2.29	46.95
17	2.43	3.43	1.87	0.60	2.26	49.21
59	2.98	2.41	1.80	0.61	2.18	51.39
26	7.20	0.00	1.69	0.86	2.05	53.44
69	6.31	0.00	1.65	0.70	1.99	55.43
11	1.99	2.29	1.33	0.55	1.61	57.04
501	2.91	0.00	1.27	0.36	1.53	58.58
22	7.02	0.00	1.18	0.33	1.43	60.00
6	6.70	0.00	1.08	0.62	1.31	61.31
10	2.94	0.85	1.05	0.55	1.27	62.58
24	1.58	1.12	1.03	0.49	1.25	63.83
30	2.49	1.14	1.01	0.50	1.23	65.06
52	4.11	0.00	1.00	0.82	1.21	66.26
36	3.65	0.00	0.95	0.73	1.15	67.42
702	5.31	0.00	0.87	0.46	1.06	68.47
362	0.00	1.99	0.72	0.29	0.87	69.34
464	2.52	0.00	0.67	0.49	0.81	70.15

12	3.75	0.00	0.63	0.33	0.76	70.91
31	2.46	0.00	0.58	0.50	0.70	71.62
265	1.11	0.00	0.57	0.33	0.69	72.30
27	3.01	0.00	0.51	0.33	0.61	72.91
801	3.41	0.00	0.50	0.44	0.61	73.52
771	2.78	0.00	0.47	0.43	0.56	74.09
796	1.76	0.00	0.46	0.33	0.56	74.64
363	0.00	1.25	0.45	0.29	0.55	75.19
760	1.69	0.00	0.44	0.33	0.53	75.72
468	0.00	1.30	0.44	0.29	0.53	76.25
19	2.55	0.00	0.43	0.33	0.52	76.77
817	2.83	0.00	0.42	0.48	0.51	77.28
266	0.78	0.00	0.40	0.33	0.48	77.76
675	0.72	0.87	0.40	0.36	0.48	78.24
29	0.77	0.00	0.39	0.33	0.48	78.71
745	2.27	0.00	0.39	0.50	0.48	79.19
654	0.96	0.00	0.39	0.33	0.47	79.66
724	1.45	0.00	0.38	0.33	0.46	80.12
409	0.00	1.00	0.36	0.29	0.44	80.56
810	2.29	0.00	0.35	0.49	0.42	80.98
815	2.45	0.00	0.34	0.33	0.41	81.39
693	1.44	0.00	0.31	0.48	0.37	81.77
762	2.22	0.00	0.31	0.33	0.37	82.14
791	1.66	0.00	0.30	0.33	0.36	82.50
658	0.73	0.00	0.30	0.33	0.36	82.86
565	0.72	0.00	0.29	0.33	0.35	83.21
45	0.73	0.00	0.29	0.33	0.35	83.56
783	1.59	0.00	0.29	0.33	0.35	83.91
708	1.04	0.00	0.27	0.33	0.33	84.24
811	1.50	0.00	0.27	0.33	0.33	84.56
758	1.60	0.00	0.27	0.33	0.33	84.89
785	1.49	0.00	0.27	0.33	0.32	85.21
735	1.02	0.00	0.27	0.33	0.32	85.54
725	1.70	0.00	0.26	0.50	0.31	85.85
731	0.97	0.00	0.26	0.33	0.31	86.16
670	1.51	0.00	0.25	0.33	0.31	86.46
728	1.63	0.00	0.25	0.50	0.30	86.76
617	0.90	0.00	0.24	0.33	0.28	87.05
765	0.89	0.00	0.23	0.33	0.28	87.33
763	1.30	0.00	0.23	0.33	0.28	87.61
614	0.88	0.00	0.23	0.33	0.28	87.89
68	1.32	0.00	0.22	0.33	0.27	88.16
761	1.23	0.00	0.22	0.33	0.27	88.42
640	0.84	0.00	0.22	0.33	0.26	88.69
721	0.82	0.00	0.22	0.33	0.26	88.95
656	0.81	0.00	0.21	0.33	0.26	89.21
491	0.77	0.00	0.20	0.33	0.25	89.45
808	0.77	0.00	0.20	0.33	0.24	89.69
786	1.44	0.00	0.20	0.33	0.24	89.94
701	0.75	0.00	0.20	0.33	0.24	90.18

Groups 1 & 7
Average dissimilarity = 77.84

	Group 1	Group 7				
Species	Av.Abund	Av.Abund	Av.Diss	Diss/SD	Contrib%	Cum.%
8	23.09	33.36	9.36	1.02	12.03	12.03
16	14.99	12.07	5.42	0.97	6.96	18.99
25	0.00	14.79	5.15	1.42	6.62	25.61
23	19.00	7.68	4.13	1.10	5.30	30.91
5	6.94	0.00	3.89	0.70	5.00	35.91
6	11.45	1.96	3.43	0.87	4.41	40.32
26	11.93	0.00	3.31	1.00	4.25	44.57
21	5.37	8.75	3.01	1.01	3.87	48.43

9	0.00	8.73	2.96	0.45	3.81	52.24
11	10.11	0.00	2.77	0.80	3.56	55.80
17	0.00	4.71	2.00	0.46	2.57	58.38
464	7.30	0.72	1.92	0.50	2.47	60.84
10	0.00	4.78	1.62	0.46	2.08	62.92
7	3.07	0.00	1.55	0.36	1.99	64.91
707	5.40	0.00	1.37	0.37	1.76	66.67
30	0.00	3.28	1.30	0.59	1.67	68.34
467	3.66	1.32	1.18	0.63	1.52	69.86
465	0.00	4.10	1.13	0.46	1.45	71.31
70	3.90	0.00	1.08	0.56	1.38	72.69
24	0.00	3.87	1.03	0.32	1.33	74.02
461	4.28	0.00	1.02	0.37	1.32	75.34
431	3.23	0.00	0.97	0.53	1.24	76.58
53	2.97	0.00	0.80	0.75	1.03	77.61
405	1.50	0.00	0.76	0.36	0.97	78.58
709	2.38	0.00	0.72	0.37	0.93	79.51
404	1.43	0.00	0.72	0.36	0.92	80.43
69	1.66	1.48	0.71	0.49	0.92	81.35
712	2.02	0.71	0.68	0.47	0.87	82.21
36	0.92	1.86	0.65	0.59	0.83	83.04
37	2.22	0.00	0.61	0.56	0.79	83.83
725	1.34	0.87	0.57	0.48	0.73	84.56
68	0.00	1.87	0.53	0.32	0.67	85.23
713	2.04	0.00	0.52	0.37	0.67	85.90
807	0.00	2.11	0.50	0.32	0.65	86.55
29	1.00	1.03	0.50	0.48	0.64	87.19
52	0.00	1.77	0.46	0.47	0.59	87.78
459	0.00	1.64	0.44	0.32	0.56	88.35
666	1.66	0.00	0.42	0.37	0.54	88.89
777	0.00	1.76	0.42	0.32	0.54	89.43
659	1.57	0.00	0.40	0.37	0.51	89.94
816	0.00	1.48	0.39	0.47	0.50	90.44

Groups 2 & 7
Average dissimilarity = 79.14

	Group 2	Group 7				
Species	Av.Abund	Av.Abund	Av.Diss	Diss/SD	Contrib%	Cum.%
8	17.86	33.36	13.09	0.91	16.54	16.54
25	0.00	14.79	6.34	1.25	8.01	24.55
9	6.92	8.73	5.45	0.63	6.88	31.43
16	5.92	12.07	5.22	0.68	6.60	38.03
463	16.39	0.00	4.79	0.59	6.05	44.08
21	4.81	8.75	4.15	0.84	5.25	49.33
17	2.44	4.71	2.91	0.49	3.67	53.00
23	9.64	7.68	2.63	1.03	3.33	56.32
10	0.00	4.78	2.00	0.44	2.53	58.85
30	2.15	3.28	1.81	0.64	2.29	61.15
24	1.25	3.87	1.41	0.38	1.78	62.93
6	4.81	1.96	1.38	0.63	1.75	64.68
11	3.98	0.00	1.33	0.59	1.68	66.36
465	0.00	4.10	1.29	0.45	1.63	67.98
26	5.38	0.00	1.16	0.76	1.47	69.46
491	2.14	0.00	0.98	0.36	1.24	70.69
70	4.31	0.00	0.91	0.57	1.16	71.85
460	2.90	1.21	0.89	0.50	1.12	72.97
36	2.18	1.86	0.86	0.67	1.09	74.06
789	3.14	0.96	0.85	0.63	1.07	75.13
69	1.82	1.48	0.74	0.48	0.94	76.07
706	3.75	0.00	0.74	0.37	0.93	77.00
52	0.94	1.77	0.67	0.56	0.84	77.84
461	3.08	0.00	0.65	0.56	0.82	78.66
7	2.75	0.00	0.62	0.37	0.79	79.45

68	0.00	1.87	0.60	0.31	0.76	80.21
785	2.55	0.00	0.58	0.37	0.73	80.94
807	0.00	2.11	0.56	0.32	0.71	81.64
53	2.35	0.00	0.50	0.57	0.63	82.27
459	0.00	1.64	0.50	0.31	0.63	82.90
655	2.09	0.00	0.47	0.37	0.60	83.50
777	0.00	1.76	0.47	0.32	0.59	84.09
666	1.93	0.00	0.44	0.37	0.55	84.64
816	0.00	1.48	0.44	0.46	0.55	85.19
35	0.00	1.43	0.43	0.31	0.55	85.74
800	0.99	0.91	0.43	0.48	0.54	86.29
791	0.95	0.78	0.41	0.46	0.51	86.80
467	0.00	1.32	0.40	0.31	0.50	87.30
717	1.74	0.00	0.39	0.37	0.50	87.80
14	0.96	0.73	0.38	0.48	0.48	88.28
358	0.00	1.18	0.36	0.31	0.45	88.73
29	0.00	1.03	0.33	0.31	0.42	89.15
22	1.42	0.00	0.32	0.37	0.41	89.56
796	1.63	0.00	0.32	0.37	0.41	89.97
792	0.00	1.14	0.30	0.32	0.38	90.35

Groups 3 & 7
Average dissimilarity = 76.05

	Group 3	Group 7				
Species	Av.Abund	Av.Abund	Av.Diss	Diss/SD	Contrib%	Cum.%
8	14.46	33.36	14.56	1.16	19.15	19.15
25	0.00	14.79	7.59	1.57	9.99	29.13
16	4.29	12.07	6.75	0.85	8.88	38.01
9	7.68	8.73	6.59	0.69	8.67	46.68
21	7.85	8.75	6.03	1.15	7.93	54.62
23	7.12	7.68	4.06	0.96	5.34	59.95
17	0.00	4.71	3.13	0.48	4.12	64.08
6	4.48	1.96	2.66	0.61	3.50	67.58
11	4.05	0.00	2.58	0.65	3.39	70.97
10	0.00	4.78	2.39	0.48	3.15	74.12
30	0.00	3.28	1.98	0.63	2.61	76.73
24	1.18	3.87	1.98	0.46	2.60	79.33
465	0.00	4.10	1.52	0.48	2.00	81.33
7	2.50	0.00	1.45	0.36	1.90	83.23
36	1.14	1.86	0.96	0.60	1.26	84.49
12	2.56	0.00	0.96	0.37	1.26	85.75
26	2.21	0.00	0.83	0.37	1.09	86.84
68	0.00	1.87	0.71	0.33	0.94	87.77
807	0.00	2.11	0.65	0.33	0.86	88.63
52	0.00	1.77	0.62	0.48	0.81	89.44
459	0.00	1.64	0.58	0.33	0.77	90.21

Groups 4 & 7
Average dissimilarity = 71.75

	Group 4	Group 7				
Species	Av.Abund	Av.Abund	Av.Diss	Diss/SD	Contrib%	Cum.%
8	23.15	33.36	10.99	0.97	15.31	15.31
25	1.54	14.79	6.33	1.37	8.82	24.13
16	9.54	12.07	6.31	0.91	8.79	32.92
9	2.57	8.73	4.87	0.57	6.79	39.71
21	4.87	8.75	4.09	0.97	5.70	45.41
23	7.40	7.68	3.52	1.11	4.91	50.32
6	8.54	1.96	3.33	0.83	4.65	54.96
17	1.46	4.71	3.17	0.57	4.42	59.38
467	5.18	1.32	2.23	0.71	3.10	62.49
10	0.00	4.78	2.07	0.47	2.88	65.37

30	0.82	3.28	1.81	0.67	2.53	67.90
24	2.17	3.87	1.77	0.47	2.46	70.36
465	1.12	4.10	1.56	0.55	2.18	72.53
69	3.84	1.48	1.38	0.61	1.93	74.46
36	2.05	1.86	1.00	0.69	1.40	75.86
52	2.04	1.77	0.97	0.69	1.36	77.21
1	2.39	0.00	0.96	0.34	1.34	78.56
15	1.95	0.00	0.79	0.34	1.10	79.65
656	1.69	0.00	0.76	0.34	1.05	80.71
5	1.70	0.00	0.69	0.34	0.95	81.66
59	1.66	0.00	0.67	0.34	0.93	82.59
68	0.00	1.87	0.63	0.32	0.88	83.47
29	1.01	1.03	0.61	0.47	0.85	84.33
807	0.00	2.11	0.59	0.33	0.82	85.15
459	0.00	1.64	0.52	0.32	0.73	85.88
11	1.27	0.00	0.51	0.34	0.71	86.59
777	0.00	1.76	0.49	0.33	0.69	87.28
725	0.98	0.87	0.47	0.47	0.65	87.93
816	0.00	1.48	0.46	0.48	0.64	88.57
35	0.00	1.43	0.46	0.32	0.64	89.21
26	1.56	0.00	0.42	0.35	0.58	89.79
460	0.00	1.21	0.39	0.32	0.54	90.33

Groups 5 & 7
Average dissimilarity = 79.23

	Group 5	Group 7				
Species	Av.Abund	Av.Abund	Av.Diss	Diss/SD	Contrib%	Cum.%
8	16.80	33.36	8.37	1.05	10.56	10.56
16	12.09	12.07	4.88	0.77	6.15	16.72
9	9.27	8.73	4.39	0.74	5.54	22.25
25	1.59	14.79	4.15	1.18	5.24	27.50
21	13.10	8.75	3.42	0.94	4.31	31.81
465	7.01	4.10	2.25	0.73	2.84	34.65
17	2.43	4.71	2.09	0.54	2.63	37.29
23	8.19	7.68	2.04	0.86	2.58	39.86
10	2.94	4.78	1.81	0.57	2.28	42.15
1	5.06	0.00	1.60	0.49	2.02	44.17
467	4.68	1.32	1.58	0.46	1.99	46.16
69	6.31	1.48	1.48	0.76	1.86	48.03
26	7.20	0.00	1.41	0.89	1.79	49.81
24	1.58	3.87	1.34	0.46	1.69	51.50
30	2.49	3.28	1.32	0.67	1.66	53.17
7	5.55	0.00	1.30	0.58	1.64	54.81
6	6.70	1.96	1.25	0.68	1.58	56.39
15	3.45	0.00	1.09	0.49	1.38	57.77
5	3.43	0.00	1.09	0.49	1.37	59.14
22	7.02	0.00	1.05	0.33	1.33	60.47
36	3.65	1.86	0.98	0.82	1.24	61.71
52	4.11	1.77	0.98	0.88	1.24	62.95
501	2.91	0.00	0.96	0.36	1.21	64.15
59	2.98	0.00	0.94	0.49	1.19	65.35
702	5.31	0.00	0.78	0.46	0.99	66.33
464	2.52	0.72	0.66	0.57	0.83	67.16
68	1.32	1.87	0.63	0.40	0.79	67.95
12	3.75	0.00	0.56	0.33	0.71	68.66
11	1.99	0.00	0.53	0.48	0.67	69.33
29	0.77	1.03	0.51	0.44	0.64	69.97
31	2.46	0.00	0.49	0.49	0.62	70.59
35	1.05	1.43	0.47	0.41	0.59	71.18
801	3.41	0.00	0.46	0.44	0.57	71.75
27	3.01	0.00	0.45	0.33	0.57	72.33
807	0.00	2.11	0.45	0.31	0.56	72.89
791	1.66	0.78	0.44	0.46	0.55	73.44

265	1.11	0.00	0.43	0.32	0.54	73.98
771	2.78	0.00	0.42	0.43	0.52	74.50
460	1.02	1.21	0.39	0.41	0.50	75.00
796	1.76	0.00	0.39	0.33	0.49	75.49
459	0.00	1.64	0.39	0.31	0.49	75.98
19	2.55	0.00	0.38	0.33	0.48	76.46
817	2.83	0.00	0.38	0.48	0.48	76.94
725	1.70	0.87	0.38	0.55	0.48	77.42
760	1.69	0.00	0.37	0.33	0.47	77.89
777	0.00	1.76	0.37	0.31	0.47	78.36
745	2.27	0.00	0.35	0.49	0.44	78.80
816	0.00	1.48	0.34	0.45	0.43	79.23
724	1.45	0.00	0.32	0.33	0.41	79.64
810	2.29	0.00	0.32	0.48	0.40	80.04
815	2.45	0.00	0.31	0.33	0.39	80.43
654	0.96	0.00	0.31	0.33	0.39	80.81
266	0.78	0.00	0.30	0.32	0.38	81.19
762	2.22	0.00	0.28	0.33	0.35	81.54
358	0.00	1.18	0.28	0.31	0.35	81.90
693	1.44	0.00	0.27	0.48	0.34	82.23
783	1.59	0.00	0.25	0.33	0.32	82.55
792	0.00	1.14	0.24	0.31	0.30	82.86
758	1.60	0.00	0.24	0.33	0.30	83.16
811	1.50	0.00	0.24	0.33	0.30	83.46
814	0.00	1.12	0.24	0.31	0.30	83.76
785	1.49	0.00	0.24	0.33	0.30	84.06
658	0.73	0.00	0.23	0.33	0.30	84.36
708	1.04	0.00	0.23	0.33	0.29	84.65
772	0.00	1.09	0.23	0.31	0.29	84.94
565	0.72	0.00	0.23	0.33	0.29	85.23
45	0.73	0.00	0.23	0.33	0.29	85.52
670	1.51	0.00	0.23	0.33	0.29	85.81
735	1.02	0.00	0.23	0.33	0.29	86.09
728	1.63	0.00	0.22	0.49	0.28	86.37
731	0.97	0.00	0.22	0.33	0.27	86.65
763	1.30	0.00	0.21	0.33	0.26	86.91
789	0.00	0.96	0.20	0.31	0.26	87.16
617	0.90	0.00	0.20	0.33	0.25	87.42
765	0.89	0.00	0.20	0.33	0.25	87.66
761	1.23	0.00	0.20	0.33	0.25	87.91
614	0.88	0.00	0.19	0.33	0.25	88.16
800	0.00	0.91	0.19	0.31	0.24	88.40
640	0.84	0.00	0.19	0.33	0.23	88.63
726	0.00	0.87	0.18	0.31	0.23	88.86
721	0.82	0.00	0.18	0.33	0.23	89.09
509	0.00	0.73	0.18	0.31	0.23	89.32
786	1.44	0.00	0.18	0.33	0.23	89.55
656	0.81	0.00	0.18	0.33	0.23	89.78
804	1.39	0.00	0.18	0.33	0.22	90.00

Groups 6 & 7
Average dissimilarity = 73.52

	Group 6	Group 7				
Species	Av.Abund	Av.Abund	Av.Diss	Diss/SD	Contrib%	Cum.%
8	16.18	33.36	13.09	1.39	17.81	17.81
25	1.44	14.79	7.02	1.51	9.54	27.35
9	5.57	8.73	6.20	0.72	8.43	35.78
16	0.00	12.07	5.61	0.69	7.64	43.41
21	4.30	8.75	4.56	1.09	6.20	49.61
17	3.43	4.71	4.01	0.71	5.46	55.07
23	6.91	7.68	3.74	1.01	5.09	60.17
10	0.85	4.78	2.59	0.56	3.53	63.70
467	4.58	1.32	2.33	0.68	3.17	66.86

30	1.14	3.28	2.18	0.70	2.97	69.84
24	1.12	3.87	1.91	0.45	2.60	72.44
465	1.07	4.10	1.82	0.58	2.48	74.92
59	2.41	0.00	1.16	0.46	1.58	76.50
11	2.29	0.00	1.12	0.46	1.52	78.02
15	2.33	0.00	1.08	0.48	1.47	79.49
5	2.30	0.00	1.08	0.48	1.47	80.97
362	1.99	0.00	0.88	0.32	1.19	82.16
68	0.00	1.87	0.70	0.33	0.95	83.11
807	0.00	2.11	0.64	0.33	0.87	83.98
36	0.00	1.86	0.63	0.48	0.86	84.84
7	1.13	0.00	0.62	0.32	0.84	85.68
52	0.00	1.77	0.60	0.48	0.82	86.50
6	0.00	1.96	0.60	0.33	0.81	87.31
459	0.00	1.64	0.57	0.33	0.78	88.09
363	1.25	0.00	0.55	0.32	0.75	88.84
777	0.00	1.76	0.53	0.33	0.73	89.57
468	1.30	0.00	0.53	0.32	0.72	90.29
