# Supplementary material for: Maize Phyllosphere Microbial Community Niche Development Across Stages of Host Leaf Growth
Source: F1000Res. 2018 Jan 18;6:1698. Originally published 2017 Sep 18. [Version 3] doi: 10.12688/f1000research.12490.3 (PMC5861518; doi:10.12688/f1000research.12490.3)
Supplement: Supplementary file 6 [file f1000research-6-14916-s0009.tgz › 08ad5d61-0bb0-4f7b-9ce8-26f4920cac90.doc]

S1Results = Supplemental_Results_OTU_SCU_Distributions

The full JMP output of the nonparametric analysis of means across plant stages for diversity measures is provided below for each microbial community measurement type. Distributions for SEM SCU by stage are also provided.

ARISA:

a. Analysis of S (total species = total OTU)

b. Analysis of Pielou’s evenness (J’). Note that the ANOM test was manually adjusted for multiple testing, while the pairwise tests are adjusted for multiple testing within the procedure.

SEM:

a. Total species (S) = total SCU

b. Analysis of evenness, J’

c. Distribution of SEM sizeclass areas. The area size unit is micrometers squared.
