# Supplementary material for: Maize Phyllosphere Microbial Community Niche Development Across Stages of Host Leaf Growth
Source: F1000Res. 2018 Jan 18;6:1698. Originally published 2017 Sep 18. [Version 3] doi: 10.12688/f1000research.12490.3 (PMC5861518; doi:10.12688/f1000research.12490.3)
Supplement: Peer review history

# Peer review: Referee response for version 3

**DOI:** 10.5256/f1000research.14916.r31264

**Published:** 2018-03-20

**Authors:** Kiwamu Minamisawa

**Affiliations:** 1 Graduate School of Life Sciences, Tohoku University, Sendai, Japan

This manuscript describes that taxonomic and cell-size patterns provided evidence for niche-based contributions to microbial community development on maize leaves across host leaf stages. Although the cell-size trait patterns by SEM for evaluations of microbial communities looks really novel in this area, the criteria for the cell-size trait are not fully described in this manuscript. I recommended that the authors more clearly explain the criteria for the cell-size trait in the text for readers who want to follow the methodology and criteria. From Figure 2, there are many shapes and length of bacterial cells on the surface of maize leaves. However, they were transformed ranks in Fig. 3B and Fig. 5, which were likely classified into size-class unit (SCU) s1~s35 through size-class unit (SCU) by SEM image analysis. Can the authors present the 35 SCUs for readers in the text?  Are the SCU determinations based on both cell size and cell shapes (rod or coccus)? I believe that these efforts and explanations would improve the quality of this manuscript for more broad readers including microbiologists beyond many technical arguments.

I have read this submission. I believe that I have an appropriate level of expertise to confirm that it is of an acceptable scientific standard.

# Peer review: Referee response for version 3

**DOI:** 10.5256/f1000research.14916.r30003

**Published:** 2018-01-25

**Authors:** Mitja N.P. Remus-Emsermann, Erica Boyd, Michal Bernach, Rudolf Schlechter, Simisola Oso

**Affiliations:** 1 School of Biological Sciences, University of Canterbury, Christchurch, New Zealand

I agree with the revisions.

We have read this submission. We believe that we have an appropriate level of expertise to confirm that it is of an acceptable scientific standard.

# Peer review: Referee response for version 2

**DOI:** 10.5256/f1000research.14470.r28298

**Published:** 2018-01-02

**Authors:** Mitja N.P. Remus-Emsermann, Michal Bernach, Rudolf Schlechter, Erica Boyd, Simisola Oso

**Affiliations:** 1 School of Biological Sciences, University of Canterbury, Christchurch, New Zealand

In the study described in this article, the authors use two complementary approachers to investigate the bacterial diversity on corn leaves over time. They aimed at untangling the bacterial community assembly processes on corn leaves. The authors were the first to my knowledge to investigate temporal community development on corn plants. 

 The molecular work is thorough, discussed well and leaves little room for improvement based on the techniques employed. However, I have major objections about the use of scanning electron microscopy as a tool for community investigations. I keep but wondering why the authors tried to use it in the first place since a literature search would have revealed that microscopy without molecular tools is not suitable for community investigations. This is why I propose to stress early on in the manuscript that this is a failed attempt to combine DNA-based molecular techniques and morphological data.

 The following paragraph reads somewhat abrupt but they entail my concerns about the microscopical analysis and how they may be improved in the future.

 The analysis of electron microscopical pictures is highly problematic as debris will be virtually and practically indistinguishable from bacteria at the resolution the images were acquired. Even a well trained analyst will not be able to distinguish them. Additionally, yeast will be visible in those images that would not be picked up in the ARISA analysis. Furthermore, the preparation of the specimen will remove bacterial biomass from leaves. The analysis of the SEM pictures that was performed manually instead of using computer-based thresholds or filters, even though using trained individuals, may also be highly problematic to determine the size and shape of individual bacteria since the ability of the analyst to surround the bacterial cell is limited by their ability to draw using a mouse/ touchpad etc.. This is well visible on the right hand side of Figure 2. The abundance of bacterial biomass seems extremely low - it would help the reader to provide a graphical representation of the biomass coverage and its development over time.  I recommend the repeat the experiment using an independent marker such as a DNA intercalating fluorescent dye or fluorescence in situ hybridisation and fluorescence microscopy. Since spatial data was not important to the analysis, I also recommend to recover the bacterial biomass by washing instead of performing on leaf observations. In general however,  I am very doubtful of the use of size markers to cluster bacterial cells into operational taxonomic unit equivalents, bacteria are notoriously flexible if it comes to size: imagine a cell that is growing up to soon divide versus a cell that freshly divided. Frank Dazzo's work, which is also cited by the authors takes the cell size analysis a step further and includes additional geometrical measures to determine "morphotypes" that he uses as a proxy for diversity, which is something that the authors should consider to improve the resolution of their SCU analysis. I understand however, that there is no suitable technique available thus far that would yield a single-cell result that would resemble an OTU-based analysis. A combination of FISH + morphological analysis may be the most advanced option thus far.  In summary, I am missing a motivation in the introduction for the used technique and why the authors believed that is would offer merit over the complementary ARISA approach.

 From the current description it is unclear to me how different SCUs were assigned, how many classes were detected etc. Some corresponding results are well hidden in the supplemental data (S1 C), however in the corresponding graphs, the units are lacking. My guess is, that they refer to µmˆ2, then again, I am confused that the chosen bins are of different width each time. Again surprising to me is, that some identified bacteria projected an area as large as 20 µmˆ2, which seems unlikely. As a side note, the quality of the pictures is very low and they are full of compression artefacts, the authors should provide a higher quality screenshot to aid readability. 

 Additional discussion why image analysis was not suitable and how it may be improved in the future would be useful.

 Other general comments:

 I believe that the more recent works of Steve Kembel should be cited e.g. Tree phyllosphere bacterial communities: exploring the magnitude of intra- and inter-individual variation among host species ^1^.

Host species identity, site and time drive temperate tree phyllosphere bacterial community structure. Isabelle Laforest-Lapointe, Christian Messier and Steven W. Kembel ^2^.

 As well as: Maignien L, DeForce EA, Chafee ME, Eren AM, Simmons SL. 2014. Ecological succession and stochastic variation in the assembly of Arabidopsis thaliana phyllosphere communities ^3^.

 The authors focus their discussion almost exclusively on Shade et al. 2013 ^4^. Shade et al investigated the very short-lived microbiome “flower” while in the presented study relatively long lived leaves are under investigation. The citations above should provide additional important material for the discussion and should provide data from habitats that are a better comparison than flowers.

 Specific comments: 

 The first sentence of the introduction should be corrected as the phyllosphere is the habitat that is colonised by microbial communities, not the community itself.

 Second last paragraph of the introduction: the authors cite one of my papers to support their statement that bacteria can be tracked by using (fluorescent) reporter genes, however, in the cited paper, bacteria were washed of plant leaves prior to their investigation, therefore other papers, such as Monier and Lindow 2005 ^5^ are more suitable citations here. If the authors insist to cite me, Remus-Emsermann et al. 2011 ^6^ is more appropriate. 

 Methods:

 Electron microscopy: Instead, or additionally, to referring the absolute magnification, acquired area should be mentioned to better understand how representative the acquired field of views were.

 The "Ribosomal spacer amplification and separation (ARISA) paragraph" ends with "Bacterial sizes acquired from all images were binned using methods described by Shimazaki et al.. Bin sizes were assigned to the microbial samples as size-class units (SCU) and a microbial community profile was created for the samples using the RiboSort package in R." I assume this should be shifted to the previous paragraph, otherwise I don't understand the paragraph

 Paragraph “SEM image analysis”: it is not clear to me how Image Pro Plus was used to measure “bacterial areas”, did you employ a detection algorithm that only worked within the circled areas, or were the circled areas themselves measures?

 To maintain a parallel structure throughout the manuscript, i.e. first SEM analysis and then ARISA analysis, Table 1 and Table 2 should be switched in sequence. The same is true for Figure 3 a and b and Figure 4 and 5. Alternatively, the method sections could be switched in sequence.

 the term SEM OTU occurs in table 3 - this should be corrected to SCU. 

 Supplement S1: the description of the figures is not self explanatory and should be improved. Suggestion: “Analysis of S…” and  “Total species (S)…” should read “Taxa_S = Total OTU” or “SCU” to be consistent with the nomenclature used in the graph, or the label of the y-axis could be changed.  

 The paper was subject of a journal club in my group and members of my lab group contributed to this review.

We have read this submission. We believe that we have an appropriate level of expertise to confirm that it is of an acceptable scientific standard, however we have significant reservations, as outlined above.

## References

[1] Laforest-LapointeIMessierCKembelSW: Tree phyllosphere bacterial communities: exploring the magnitude of intra- and inter-individual variation among host species. *PeerJ*.2016;4: 10.7717/peerj.2367 e2367 10.7717/peerj.2367 27635335PMC5012278

[2] Laforest-LapointeIMessierCKembelS: Host species identity, site and time drive temperate tree phyllosphere bacterial community structure. *Microbiome*.2016;4(1) : 10.1186/s40168-016-0174-1 10.1186/s40168-016-0174-1 27316353PMC4912770

[3] MaignienLDeForceEAChafeeMEErenAMSimmonsSL: Ecological succession and stochastic variation in the assembly of Arabidopsis thaliana phyllosphere communities. *MBio*.2014;5(1) : 10.1128/mBio.00682-13 e00682-13 10.1128/mBio.00682-13 24449749PMC3903271

[4] ShadeAMcManusPSHandelsmanJ: Unexpected diversity during community succession in the apple flower microbiome. *MBio*.2013;4(2) : 10.1128/mBio.00602-12 10.1128/mBio.00602-12 23443006PMC3585449

[5] MonierJMLindowSE: Spatial organization of dual-species bacterial aggregates on leaf surfaces. *Appl Environ Microbiol*.2005;71(9) : 10.1128/AEM.71.9.5484-5493.2005 5484-93 10.1128/AEM.71.9.5484-5493.2005 16151141PMC1214633

[6] Remus-EmsermannMNde OliveiraSSchreiberLLeveauJH: Quantification of lateral heterogeneity in carbohydrate permeability of isolated plant leaf cuticles. *Front Microbiol*.2011;2: 10.3389/fmicb.2011.00197 197 10.3389/fmicb.2011.00197 22046169PMC3202220

# Response

**Published:** 2018-01-10

**Authors:** Ann Stapleton

**Affiliations:** University of North Carolina Wilmington, USA

We thank the second referee group for their comments—we are especially pleased that this was used as a learning opportunity for the lab team.  Our responses to the comments are in plain text below the italicized review text.

*Referee Report 02 Jan 2018*

*Mitja N.P. Remus-Emsermann, School of Biological Sciences, University of Canterbury, Christchurch, New Zealand*

*Michal Bernach, School of Biological Sciences, University of Canterbury, Christchurch, New Zealand*

*Rudolf Schlechter, School of Biological Sciences, University of Canterbury, Christchurch, New Zealand*

*Erica Boyd, School of Biological Sciences, University of Canterbury, Christchurch, New Zealand*

*Simisola Oso, School of Biological Sciences, University of Canterbury, Christchurch, New Zealand*

*Approved with Reservations*

*In the study described in this article, the authors use two complementary approachers to investigate the bacterial diversity on corn leaves over time. They aimed at untangling the bacterial community assembly processes on corn leaves. The authors were the first to my knowledge to investigate temporal community development on corn plants. The molecular work is thorough, discussed well and leaves little room for improvement based on the techniques employed.*

Thank you, we appreciate your endorsement of our ARISA analysis.

*However, I have major objections about the use of scanning electron microscopy as a tool for community investigations. I keep but wondering why the authors tried to use it in the first place since a literature search would have revealed that microscopy without molecular tools is not suitable for community investigations. This is why I propose to stress early on in the manuscript that this is a failed attempt to combine DNA-based molecular techniques and morphological data.*

This study is a first step toward analysis of microbial morphological phenotypes, with the goal of comparing and contrasting what we see in SEM images to the ARISA results and recommending future technological and analytical improvements.  For example, we would like future researchers develop automated size-sorting of leaf washes using a cell-sorter and in-line true multivariate analysis of metabolites and microbial genomes, in a format that would allow rapid sampling in the field without need for sample preservation.  We have added additional text to the third paragraph of the introduction to state the value of non-duplicative multiple-trait measurements more clearly.

*The following paragraph reads somewhat abrupt but they entail my concerns about the microscopical analysis and how they may be improved in the future. The analysis of electron microscopical pictures is highly problematic as debris will be virtually and practically indistinguishable from bacteria at the resolution the images were acquired. Even a well trained analyst will not be able to distinguish them.*

Our review of the older SEM literature and our initial analysis of mock and inoculated samples indicates that microbial cells have distinctive features that distinguish them from dust or soil particles in images (as Dazzo et al. exploited for their algorithm).  People are generally quite effective at pattern recognition tasks of this type, so we used manual scoring of microbial cells in images -- there are good recent examples of human pattern recognition being complementary to machine learning in radiographic images, e.g [we can supply references to this topic if needed].  We certainly agree that there will be false positives in any identification process; in our case we are comparing samples within our experiment, so as long as our false positive and false negative rates are consistent our samples can be compared to each other. 

*Additionally, yeast will be visible in those images that would not be picked up in the ARISA analysis. Furthermore, the preparation of the specimen will remove bacterial biomass from leaves. The analysis of the SEM pictures that was performed manually instead of using computer-based thresholds or filters, even though using trained individuals, may also be highly problematic to determine the size and shape of individual bacteria since the ability of the analyst to surround the bacterial cell is limited by their ability to draw using a mouse/ touchpad etc..*

*This is well visible on the right hand side of Figure 2.*

The image shown in Figure 2 is a relatively low-resolution figure; high-resolution images are available in the bisque repository (along with our manual traces, for future comparisons).  The analysis process was done on the same computer and monitor setup, so the analysts’ resolution for tracing would be the same for each sample. 

*The abundance of bacterial biomass seems extremely low - it would help the reader to provide a graphical representation of the biomass coverage and its development over time.  I recommend the repeat the experiment using an independent marker such as a DNA intercalating fluorescent dye or fluorescence in situ hybridisation and fluorescence microscopy. Since spatial data was not important to the analysis, I also recommend to recover the bacterial biomass by washing instead of performing on leaf observations. In general however,  I am very doubtful of the use of size markers to cluster bacterial cells into operational taxonomic unit equivalents, bacteria are notoriously flexible if it comes to size: imagine a cell that is growing up to soon divide versus a cell that freshly divided. Frank Dazzo's work, which is also cited by the authors takes the cell size analysis a step further and includes additional geometrical measures to determine "morphotypes" that he uses as a proxy for diversity, which is something that the authors should consider to improve the resolution of their SCU analysis. I understand however, that there is no suitable technique available thus far that would yield a single-cell result that would resemble an OTU-based analysis. A combination of FISH + morphological analysis may be the most advanced option thus far.  In summary, I am missing a motivation in the introduction for the used technique and why the authors believed that is would offer merit over the complementary ARISA approach.*

We very much agree that method developments are urgently needed in this area.  As you note, there are many interesting new image-based and sensor-based technologies that we think could be used for future studies.  Since we conclude from our results that morphological phenotypes provide additional, new information beyond what we can learn from ribotype patterns, we thus provide the background for future researchers to justify further development of morphological phenotype measurement tools.  We argue that morphology, metabolites, functional genes, proteins, and pathways are likely to be important for microbial community development and thus we encourage automated sampling device development that could measure all these traits in each sample.  Future image analyses could also use spatial modeling theory and analysis tools to examine microbial distributions along the heterogenous and rugged leaf surface, possibly with a prior that adjacent cells are more likely to be related than distant cells.  A micro-sampler that could select individual cells as it traversed the surface would be the optimal solution, though substantial technology development would be needed to create such a sampling device.

*From the current description it is unclear to me how different SCUs were assigned, how many classes were detected etc. Some corresponding results are well hidden in the supplemental data (S1 C), however in the corresponding graphs, the units are lacking. My guess is, that they refer to µmˆ2, then again, I am confused that the chosen bins are of different width each time. Again surprising to me is, that some identified bacteria projected an area as large as 20 µmˆ2, which seems unlikely. As a side note, the quality of the pictures is very low and they are full of compression artefacts, the authors should provide a higher quality screenshot to aid readability.*

*Additional discussion why image analysis was not suitable and how it may be improved in the future would be useful.*

We revised our methods section description to make it clear that these SCU refer to microbial cells, not necessarily bacteria.  We added the area units to supplemental results section c. The full list of SCU taxa with sizes and other details is available in the CyVerse data file “binnedSEMsizes.csv”.  All the images and metadata are available in the Bisque repository for future development of image analysis methods; each image has the keV, scale bar, magnification and quadrant size included within the image file.

*Other general comments:*

*I believe that the more recent works of Steve Kembel should be cited e.g. Tree phyllosphere bacterial communities: exploring the magnitude of intra- and inter-individual variation among host species* *^1^* *.*

*Host species identity, site and time drive temperate tree phyllosphere bacterial community structure* *. Isabelle Laforest-Lapointe, Christian Messier and Steven W. Kembel* *^2^* *.*

*As well as: Maignien L, DeForce EA, Chafee ME, Eren AM, Simmons SL. 2014. Ecological succession and stochastic variation in the assembly of Arabidopsis thaliana phyllosphere communities* *^3^* *.*

*The authors focus their discussion almost exclusively on Shade et al. 2013* *^4^* *. Shade et al investigated the very short-lived microbiome “flower” while in the presented study relatively long lived leaves are under investigation. The citations above should provide additional important material for the discussion and should provide data from habitats that are a better comparison than flowers.*

Maize leaves vary in lifespan over development, with the youngest leaves being shed after approximately ten days and upper leaves remaining until the end of the season (~90 days).  We have added the citations you suggest to our discussion section.

*Specific comments:*

*The first sentence of the introduction should be corrected as the phyllosphere is the habitat that is colonised by microbial communities, not the community itself.*

Corrected.

*Second last paragraph of the introduction: the authors cite one of my papers to support their statement that bacteria can be tracked by using (fluorescent) reporter genes, however, in the cited paper, bacteria were washed of plant leaves prior to their investigation, therefore other papers, such as Monier and Lindow 2005* *^5^* *are more suitable citations here. If the authors insist to cite me, Remus-Emsermann et al. 2011* *^6^* *is more appropriate.*

We have updated the citations as you suggest.

*Methods:*

*Electron microscopy: Instead, or additionally, to referring the absolute magnification, acquired area should be mentioned to better understand how representative the acquired field of views were.*

We added that information to the methods section.

*The "Ribosomal spacer amplification and separation (ARISA) paragraph" ends with "Bacterial sizes acquired from all images were binned using methods described by Shimazaki et al.. Bin sizes were assigned to the microbial samples as size-class units (SCU) and a microbial community profile was created for the samples using the RiboSort package in R." I assume this should be shifted to the previous paragraph, otherwise I don't understand the paragraph*

Corrected, thank you for identifying this typographical error.

*Paragraph “SEM image analysis”: it is not clear to me how Image Pro Plus was used to measure “bacterial areas”, did you employ a detection algorithm that only worked within the circled areas, or were the circled areas themselves measures?*

We added more explanation of the area calculation used by this software to the methods section.

*To maintain a parallel structure throughout the manuscript, i.e. first SEM analysis and then ARISA analysis, Table 1 and Table 2 should be switched in sequence. The same is true for Figure 3 a and b and Figure 4 and 5. Alternatively, the method sections could be switched in sequence.*

After we moved the misplaced paragraph that you identified above, all the SEM image information is now in one section, before the ARISA analysis section.  The data analysis sections follow.  If sub-subheadings are permitted by the publisher we would be happy to add those, to make the methods sub-sections (such as data analysis) clear.

*the term SEM OTU occurs in table 3 - this should be corrected to SCU.*

Corrected, thank you.

*Supplement S1: the description of the figures is not self explanatory and should be improved. Suggestion: “Analysis of S…” and  “Total species (S)…” should read “Taxa_S = Total OTU” or “SCU” to be consistent with the nomenclature used in the graph, or the label of the y-axis could be changed.*

Corrected.

*The paper was subject of a journal club in my group and members of my lab group contributed to this review.*

- *Is the work clearly and accurately presented and does it cite the current literature?*

*Partly*

- *Is the study design appropriate and is the work technically sound?*

*Partly*

- *Are sufficient details of methods and analysis provided to allow replication by others?*

*Partly*

- *If applicable, is the statistical analysis and its interpretation appropriate? *

*I cannot comment. A qualified statistician is required.*

- *Are all the source data underlying the results available to ensure full reproducibility?*

*Yes*

- *Are the conclusions drawn adequately supported by the results?*

*Partly*

***References***

*1. Laforest-Lapointe I, Messier C, Kembel SW: Tree phyllosphere bacterial communities: exploring the magnitude of intra- and inter-individual variation among host species.PeerJ. 2016; **4**: e2367* *PubMed Abstract* *|* *Publisher Full Text*

*2. Laforest-Lapointe I, Messier C, Kembel S: Host species identity, site and time drive temperate tree phyllosphere bacterial community structure. Microbiome. 2016; **4** (1).* *Publisher Full Text*

*3. Maignien L, DeForce EA, Chafee ME, Eren AM, Simmons SL: Ecological succession and stochastic variation in the assembly of Arabidopsis thaliana phyllosphere communities.MBio. 2014; **5** (1): e00682-13* *PubMed Abstract* *|* *Publisher Full Text*

*4. Shade A, McManus PS, Handelsman J: Unexpected diversity during community succession in the apple flower microbiome.MBio. 2013; **4** (2).* *PubMed Abstract* *|* *Publisher Full Text*

*5. Monier JM, Lindow SE: Spatial organization of dual-species bacterial aggregates on leaf surfaces.Appl Environ Microbiol. 2005; **71** (9): 5484-93* *PubMed Abstract* *|* *Publisher Full Text*

*6. Remus-Emsermann MN, de Oliveira S, Schreiber L, Leveau JH: Quantification of lateral heterogeneity in carbohydrate permeability of isolated plant leaf cuticles.Front Microbiol. 2011; **2**: 197* *PubMed Abstract* *|* *Publisher Full Text*

***Competing Interests:*** *No competing interests were disclosed.*

***We have read this submission. We believe that we have an appropriate level of expertise to confirm that it is of an acceptable scientific standard, however we have significant reservations, as outlined above.***

# Peer review: Referee response for version 1

**DOI:** 10.5256/f1000research.13524.r26099

**Published:** 2017-10-18

**Authors:** Kathryn M. Docherty

**Affiliations:** 1 Department of Biological Sciences, Western Michigan University, Kalamazoo, MI, USA

I reviewed the manuscript "Maize phyllosphere microbial community niche development across stages of host leaf growth" by Manching et al. published in *F1000Research*. I rated this article "Approved with Reservations".

**Summary**: The researchers used a staggered experimental design to examine microbial community assembly processes on leaves of *Zea mays*. That is, they planted seeds at different times, and then collected samples on one date from 7 different growth stages of maize (Days 30, 41, 48, 58, 62, 72, 80). They examined leaf samples from 10 plants at each growth stage using two approaches. First, they examined microbial communities using SEM and categorized microorganisms into different size class units (SCUs). Second, they examined microbial communities using ARISA and categorized taxa into different operational taxonomic units (OTUs). Using microscopic and molecular approaches they found different results. Size-classes varied on days 41 and 48, first dropping in average size and then increasing in average size. On the rest of the dates, the size classes did not differ. OTUs were different between days 30 and 80, but for the rest of the time period, the communities did not differ significantly. To explore this further, the researchers used a network analysis approach to elucidate how either specific SCUs or OTUs were related to changes (positive or negative) in other SCUs or OTUs, respectively. They found that there were more positive co-occurrences than negative co-occurrences in both datasets, but that most individual data points did not have a significant co-occurrence pattern with other members of the dataset. They found a relatively small number of core SCUs and OTUs across the different time points of the dataset. They suggest that this may indicate that there are a number of specialists in the maize phyllosphere microbial community, and conclude that their results provide evidence for niche-based contributions to microbial community assembly on maize leaves.

 Overall, the manuscript provides novel information about changes in phyllosphere microbial communities during development stages of an important agricultural crop. The idea that plant leaves can be used for basic studies of microbial community assembly is interesting and the use of complementary datasets (size and composition) was a good approach, because it gets at 2 aspects of the community. There were a few issues with the manuscript that I believe need to be addressed before it is acceptable.

- **Work is discussed appropriately in the context of the current literature**

No, unfortunately the literature review needs to be updated. There are several important publications in this field that should be included, and this will require a re-write of the introduction and discussion sections. 

 See:

 Redford et al. ^1^

 Kembel et al. ^2^

- **Whether suitable methods have been used**

There are several things that are unclear about the choices for the experimental design. Please address:

 - Why were the specific day-intervals and developmental stage-dates chosen? Do they relate to specific physiological processes in maize development or were they chosen at random? Explain this choice further in the methods/results.

 - Planting all seeds for a particular treatment (i.e. development stage) in the same row sets the experiment up for statistical issues that are usually dealt with using a randomized block. In the current experimental design, one can interpret the results that (for example) day 30 and day 80 plant samples differ in OTU composition simply because the 30 day plants are subject to a different environmental variable than the 80 day plants (maybe soil nitrogen or light or water). Please re-do analyses using a randomized block and provide any information to suggest that there is no gradient that may account for significant differences across treatments.   

 - The samples were collected and analyzed in 2009. At that time ARISA was a suitable method for this study, but since then, several other methods have been developed that could address the hypotheses more fully. For example, using predicted metagenomics pathways might indicate that there is a connection between community structure and cell size that was not demonstrated with ARISA. The authors need to address that this could be the case in the discussion and that further studies using these techniques could yield different results.

 I liked that the investigators included control DNA extractions. Were there any OTUs associated with the controls? How were those dealt with in ARISA processing?

 I thought that the network visualization approach was interesting, but it is really unclear what the authors are showing with Figure 5b. If it is just that the interconnections between SCUs gets really complicated, then I do not think a figure is necessary.

- **Sufficient information and source data have been provided to allow others to repeat every step of the work**

Yes, I think there is sufficient information to repeat the work.

- **The conclusions are supported by the findings.**

No, I think that there are a few things that need to be addressed in the discussion. First, please refer to the references I listed above and the articles that have cited them since, and re-think the discussion in that new framework. Second, the statistical issues of having all of 1 treatment in 1 row need to be addressed, otherwise all statistical results could be considered related to some unknown environmental gradient.

**Some minor comments:**

 The introduction is mostly about community assembly. However, to me, the really interesting thing about this study is that it is done using a major agricultural crop. Little is discussed about why it would be interesting to know about bacterial community assembly in this plant. Perhaps there is a context for plant productivity or agricultural disease that could be expanded to make the intro more specific to the study?

 I found the organization of the manuscript difficult to follow. Please reorder so that all SEM-related sections are together and all ARISA sections are together in the methods. Then follow whatever order you have chosen for the results.

 The supplementary data tables are simply raw R output. Can you summarize the raw output in a supplementary table and then provide the raw output so that it is easier for the reader to interpret?

 It would be easier to interpret Tables 1 and 2 if the significances were just included in Figure 3 instead of in a separate table. (i.e. put the letters above each of the averages shown in Figure 3 to indicate significances)

 Figure 3 - the red/green dots are difficult to see (and I am not red/green color blind, so it would be even harder for 10% of our colleagues). Please adjust these so that they are more obviously different or have different shapes.

 Supplementary Figure 3 needs a legend that identifies what each of the SCU_IDs are and more information about the graph.

 On page 9, the authors state that "In the ARISA datatype, highly variable co-occurring…". Please provide more of a definition of what you mean by "highly variable". Is this quantifiable?

 Discussion Section - there are 3 different referencing styles used. Change for consistency.

I have read this submission. I believe that I have an appropriate level of expertise to confirm that it is of an acceptable scientific standard, however I have significant reservations, as outlined above.

## References

[1] RedfordAJBowersRMKnightRLinhartYFiererN: The ecology of the phyllosphere: geographic and phylogenetic variability in the distribution of bacteria on tree leaves. *Environ Microbiol*.2010;12(11) : 10.1111/j.1462-2920.2010.02258.x 2885-93 10.1111/j.1462-2920.2010.02258.x 20545741PMC3156554

[2] KembelSWO'ConnorTKArnoldHKHubbellSPWrightSJGreenJL: Relationships between phyllosphere bacterial communities and plant functional traits in a neotropical forest. *Proc Natl Acad Sci U S A*.2014;111(38) : 10.1073/pnas.1216057111 13715-20 10.1073/pnas.1216057111 25225376PMC4183302

# Response

**Published:** 2017-12-01

**Authors:** Ann Stapleton

**Affiliations:** University of North Carolina Wilmington, USA

Reply to Reviewer 1

 Thank you for your insightful comments and suggestions!  We have addressed each point in the revised manuscript, and we explain each below, with review comments in italic and author replies in regular font.

*I reviewed the manuscript "Maize phyllosphere microbial community niche development across stages of host leaf growth" by Manching et al. published in F1000Research. I rated this article "Approved with Reservations".*

**

***Summary**: The researchers used a staggered experimental design to examine microbial community assembly processes on leaves of Zea mays. That is, they planted seeds at different times, and then collected samples on one date from 7 different growth stages of maize (Days 30, 41, 48, 58, 62, 72, 80). They examined leaf samples from 10 plants at each growth stage using two approaches. First, they examined microbial communities using SEM and categorized microorganisms into different size class units (SCUs). Second, they examined microbial communities using ARISA and categorized taxa into different operational taxonomic units (OTUs). Using microscopic and molecular approaches they found different results. Size-classes varied on days 41 and 48, first dropping in average size and then increasing in average size. On the rest of the dates, the size classes did not differ. OTUs were different between days 30 and 80, but for the rest of the time period, the communities did not differ significantly. To explore this further, the researchers used a network analysis approach to elucidate how either specific SCUs or OTUs were related to changes (positive or negative) in other SCUs or OTUs, respectively. They found that there were more positive co-occurrences than negative co-occurrences in both datasets, but that most individual data points did not have a significant co-occurrence pattern with other members of the dataset. They found a relatively small number of core SCUs and OTUs across the different time points of the dataset. They suggest that this may indicate that there are a number of specialists in the maize phyllosphere microbial community, and conclude that their results provide evidence for niche-based contributions to microbial community assembly on maize leaves.*

**

*Overall, the manuscript provides novel information about changes in phyllosphere microbial communities during development stages of an important agricultural crop. The idea that plant leaves can be used for basic studies of microbial community assembly is interesting and the use of complementary datasets (size and composition) was a good approach, because it gets at 2 aspects of the community. There were a few issues with the manuscript that I believe need to be addressed before it is acceptable. *

- ***Work is discussed appropriately in the context of the current literature***

*No, unfortunately the literature review needs to be updated. There are several important publications in this field that should be included, and this will require a re-write of the introduction and discussion sections.*

*See:*

*Redford et al.* *^1^*

*Kembel et al.* *^2^*

 These references discuss across-taxa patterns, which is not our focus (we use one genotype of the domesticated crop Zea mays), so we did not include them in our manuscript.  We have added two additional sources (Redford 2009 and Dees 2015) to the introduction section to expand our coverage of seasonal pattern literature.

- **Whether suitable methods have been used**

*There are several things that are unclear about the choices for the experimental design. Please address:*

**

*- Why were the specific day-intervals and developmental stage-dates chosen? Do they relate to specific physiological processes in maize development or were they chosen at random? Explain this choice further in the methods/results.*

 These sampling intervals were chosen to capture maize leaf developmental stages (classically described as V(1 to n) for vegetative growth); each stage occurs at intervals of approximately one week and our sampling dates reflect this timing.  We added a statement to the methods section explaining this.

*- Planting all seeds for a particular treatment (i.e. development stage) in the same row sets the experiment up for statistical issues that are usually dealt with using a randomized block. In the current experimental design, one can interpret the results that (for example) day 30 and day 80 plant samples differ in OTU composition simply because the 30 day plants are subject to a different environmental variable than the 80 day plants (maybe soil nitrogen or light or water). Please re-do analyses using a randomized block and provide any information to suggest that there is no gradient that may account for significant differences across treatments.*

 The experimental design was chosen to fit agricultural practice, specifically maize field cultivation practice.  This means that seeds are planted in rows with spacing dictated by planting equipment (30 in rows at this field station) and weed control (cultivation), fertilization, and pest control are carried out in those rows.  Our experimental seeds were planted in adjacent rows, so nested spatial models that incorporate the physical distance between plants across the plots would be an ideal model to fit.  However, spatial multivariate models with zero-inflated distributions for species counts are quite complex and we chose not to add this complexity to our model, as we have no evidence for spatial gradients within these short physical distances; for example, the soil type is the same (within the limits of our measurement instruments), as is the temperature and light regime.  Our experimental design controls for field-level effects of environmental history such as recent rainfall, but cannot and does not control for developmental history of exposure to environmental factors in a field setting.  Controlled environment chambers would be required to control for past environment effects; however, chamber experiments are much less agronomically relevant, so we chose to carry out field experiments.

*- The samples were collected and analyzed in 2009. At that time ARISA was a suitable method for this study, but since then, several other methods have been developed that could address the hypotheses more fully. For example, using predicted metagenomics pathways might indicate that there is a connection between community structure and cell size that was not demonstrated with ARISA. The authors need to address that this could be the case in the discussion and that further studies using these techniques could yield different results.*

 We agree that functional gene and metabolic pathway information would be valuable, and we suggest some specific hypotheses to test using such information in the discussion section.  An automated cell-sorting assay would be required to collect suitable multivariate paired metabolic/pathway and cell size data, and we encourage development of instruments of this type.

*I liked that the investigators included control DNA extractions. Were there any OTUs associated with the controls? How were those dealt with in ARISA processing?*

 After optimization of the nested PCR protocol there were no OTU associated with the controls.  We did find that the PCR master mix was important for consistent amplification and low false positive rates, so we included details of the PCR reactions within our methods section.

*I thought that the network visualization approach was interesting, but it is really unclear what the authors are showing with Figure 5b. If it is just that the interconnections between SCUs gets really complicated, then I do not think a figure is necessary.*

 With assistance from the F1000 editorial staff we attempted to create interactive network graphics that allow readers to zoom and highlight, but it was not technically feasible at this time.  Future analyses of succession might explore network modularity measures, as the networks we see in Fig. 5 appear to show modularity, especially in conjunction with future metabolic pathway analyses.

- ***Sufficient information and source data have been provided to allow others to repeat every step of the work***

*Yes, I think there is sufficient information to repeat the work. *

- ***The conclusions are supported by the findings.***

*No, I think that there are a few things that need to be addressed in the discussion. First, please refer to the references I listed above and the articles that have cited them since, and re-think the discussion in that new framework. Second, the statistical issues of having all of 1 treatment in 1 row need to be addressed, otherwise all statistical results could be considered related to some unknown environmental gradient.*

**Some minor comments:**

*The introduction is mostly about community assembly. However, to me, the really interesting thing about this study is that it is done using a major agricultural crop. Little is discussed about why it would be interesting to know about bacterial community assembly in this plant. Perhaps there is a context for plant productivity or agricultural disease that could be expanded to make the intro more specific to the study?*

 We added more text to the first paragraph of the introduction to incorporate agricultural context.  We are pleased that you find this interesting!  There is currently substantial investment in the business sector around plant microbiomes and probiotics (several hundred million dollars, and at least four new companies that we know of), but there is relatively little publicly available basic research on agricultural plant microbiomes.  We added a citation from one review of this area (Parnell et al., 2016).

*I found the organization of the manuscript difficult to follow. Please reorder so that all SEM-related sections are together and all ARISA sections are together in the methods. Then follow whatever order you have chosen for the results.*

 We moved the SEM image analysis section to the correct place after the SEM image acquisition section in the methods.  Thank you for pointing this out!

*The supplementary data tables are simply raw R output. Can you summarize the raw output in a supplementary table and then provide the raw output so that it is easier for the reader to interpret?*

 These are output from the Primer-E program with full details of the model fits and outcomes.  We added more explanation to the methods for Tables 1 to 4 to make this clear.

*It would be easier to interpret Tables 1 and 2 if the significances were just included in Figure 3 instead of in a separate table. (i.e. put the letters above each of the averages shown in Figure 3 to indicate significances)*

 The permdisp/permanova and non-parametric richness analyses have different assumptions, so we chose not to place them in the same figure.

*Figure 3 - the red/green dots are difficult to see (and I am not red/green color blind, so it would be even harder for 10% of our colleagues). Please adjust these so that they are more obviously different or have different shapes.*

 We changed these to different colors (manually).  We agree that these are not correctly formatted and we have submitted a request to the company that developed the program asking them to adjust their defaults to color-blind-compatible colors and shapes.

*Supplementary Figure 3 needs a legend that identifies what each of the SCU_IDs are and more information about the graph.*

 We have added the caption to this figure.

*On page 9, the authors state that "In the ARISA datatype, highly variable co-occurring…". Please provide more of a definition of what you mean by "highly variable". Is this quantifiable?*

 We added more explanation to this sentence. Since network interaction patterns are a summary measure (and thus not replicated) we do not discuss them quantitatively.

*Discussion Section - there are 3 different referencing styles used. Change for consistency.*

 Updated to the F1000 style.

- Is the work clearly and accurately presented and does it cite the current literature?

No

- Is the study design appropriate and is the work technically sound?

Partly

- Are sufficient details of methods and analysis provided to allow replication by others?

Yes

- If applicable, is the statistical analysis and its interpretation appropriate?

No

- Are all the source data underlying the results available to ensure full reproducibility?

Yes

- Are the conclusions drawn adequately supported by the results?

Partly

**References**

 1. Redford AJ, Bowers RM, Knight R, Linhart Y, Fierer N: The ecology of the phyllosphere: geographic and phylogenetic variability in the distribution of bacteria on tree leaves. *Environ Microbiol*. 2010; **12** (11): 2885-93 PubMed Abstract | Publisher Full Text

 2. Kembel SW, O'Connor TK, Arnold HK, Hubbell SP, Wright SJ, Green JL: Relationships between phyllosphere bacterial communities and plant functional traits in a neotropical forest. *Proc Natl Acad Sci U S A*. 2014; **111** (38): 13715-20 PubMed Abstract | Publisher Full Text

**Competing Interests:** No competing interests were disclosed.

**Referee Expertise:** Microbial ecology, soil, airborne, biodegradation

**I have read this submission. I believe that I have an appropriate level of expertise to confirm that it is of an acceptable scientific standard, however I have significant reservations, as outlined above.**
